# Current Proteomic and Metabolomic Knowledge of Zygotic and Somatic Embryogenesis in Plants

**DOI:** 10.3390/ijms222111807

**Published:** 2021-10-30

**Authors:** Janet Juarez-Escobar, Esaú Bojórquez-Velázquez, Jose M. Elizalde-Contreras, José A. Guerrero-Analco, Víctor M. Loyola-Vargas, Martín Mata-Rosas, Eliel Ruiz-May

**Affiliations:** 1 Red de Estudios Moleculares Avanzados, Instituto de Ecología A.C., Cluster BioMimic®, Carretera Antigua a Coatepec 351, El Haya, Xalapa CP 91073, Veracruz, Mexico; janet.juarez@posgrado.ecologia.edu.mx (J.J.-E.); esau.bojorquez@inecol.mx (E.B.-V.); jose.elizalde@inecol.mx (J.M.E.-C.); 2 Unidad de Bioquímica y Biología Molecular de Plantas, Centro de Investigación Científica de Yucatán, Mérida CP 97205, Yucatán, Mexico; joseantonio.guerrero@inecol.mx (J.A.G.-A.); vmloyola@cicy.mx (V.M.L.-V.); 3 Red de Manejo Biotecnológico de Recursos, Instituto de Ecología A.C., Cluster BioMimic®, Carretera Antigua a Coatepec 351, El Haya, Xalapa CP 91073, Veracruz, Mexico; martin.mata@inecol.mx

**Keywords:** metabolomics, proteomics, somatic embryogenesis, zygotic embryogenesis, gymnosperms, angiosperms

## Abstract

Embryogenesis is the primary developmental program in plants. The mechanisms that underlie the regulation of embryogenesis are an essential research subject given its potential contribution to mass in vitro propagation of profitable plant species. Somatic embryogenesis (SE) refers to the use of in vitro techniques to mimic the sexual reproduction program known as zygotic embryogenesis (ZE). In this review, we synthesize the current state of research on proteomic and metabolomic studies of SE and ZE in angiosperms (monocots and dicots) and gymnosperms. The most striking finding was the small number of studies addressing ZE. Meanwhile, the research effort focused on SE has been substantial but disjointed. Together, these research gaps may explain why the embryogenic induction stage and the maturation of the somatic embryo continue to be bottlenecks for efficient and large-scale regeneration of plants. Comprehensive and integrative studies of both SE and ZE are needed to provide the molecular foundation of plant embryogenesis, information which is needed to rationally guide experimental strategies to solve SE drawbacks in each species.

## 1. Introduction

Embryogenesis is a critical stage in plant development, in which the single-celled zygote undergoes a polarization process and a succession of cell divisions based on cell-to-cell communication to generate a complex patterning. Patterning positions will define the fate of each cell to consequently establish the body plan necessary for vegetative growth, survival, and reproduction [1]. However, embryogenesis is driven by complex regulatory mechanisms, and the molecular and biochemical basis of the zygotic embryogenesis (ZE) process is still not entirely understood since during the early stages of ZE the zygotic embryo is embedded in maternal tissue and difficult to access [2]. Plant embryogenesis can be better defined as a consolidated event with constant changes over time, rather than a precise series of distinct events. The embryogenesis program, like many events during development, is commonly guided by gene expression, inducing changes in growth and shape, protein synthesis and molecular signaling. At the morphohistological level, embryogenesis sensu stricto is completed once the basic elements of the embryo are set.

**Figure 1:**
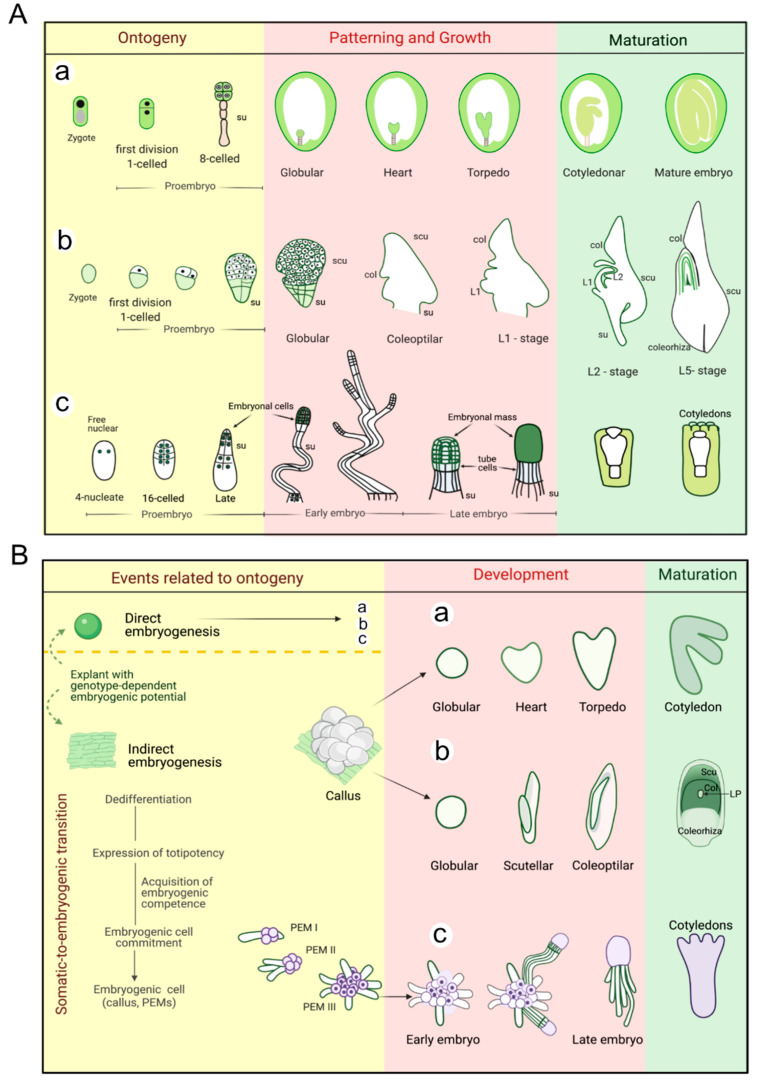
Visual representation of plant embryogenesis. Schematic representation of stereotypical morphological stages of zygotic embryo development in angiosperms and gymnosperms (**A**) Dicots of the Onagrad (*Brassicaceae)* type in *Arabidopsis thaliana* (**a**); monocots based on maize (*Poaceae*, (**b**) and gymnosperms (conifers, (**c**)). Embryo structures are not drawn to scale. Cells with dark nuclei at early stages contribute to the embryo, while cells without drawn nuclei contribute to the suspensor. Proembryo formation in dicots comprises polarity specification and formation of the suspensor and embryo proper; in monocots the suspensor region arises from irregular cell divisions of the three-celled structure; and in gymnosperms, it comprises the divisions before the suspensor elongation. Embryo development involves embryonic tissue and organ system establishment at the globular to heart-torpedo transition, or globular to scutellar stage, in dicots and monocots respectively (in nongrass monocots, the embryo has neither the coleoptile nor the coleorhiza); and the suspensor elongation and polar meristems formation in gymnosperms. Col, coleoptile; L, leaf; scu, scutellum; su, suspensor. (Own adaptation from [15,16,17]). Schematic representation of common morphological stages of somatic embryogenesis in angiosperms and gymnosperms (**B**). dicots, shown for *A. thaliana* (**a**); monocots shown for *Z. mays* (**b**)*;* gymnosperms (**c**), shown for Norway spruce. In b, leaf primordia (LP); in c, proembryogenic masses (PEMs). The embryo structures are not drawn to scale. We show the major molecular events needed to achieve the somatic-to-embryogenic cell transition which also represent the most frequently faced problems during the induction and proliferation phase, while during maturation it is the programming of the somatic embryo conversion potential to plantlet.

Most of the knowledge of angiosperm embryogenesis, specifically among Dicotyledonae (dicots) is based on studies of *Arabidopsis thaliana*, which follows a highly regular and predictable pattern of cell division and ontogeny [3,4,5]. Therefore, our discussion regarding dicotyledonous species draws on the essential patterning process in *A. thaliana*. We have also included in the review relevant information from monocots and gymnosperm species when available. In all of these groups, zygotic embryo development can be divided into three stages: (1) histodifferentiation, during which the proembryo is formed (ontogeny); (2) embryo patterning and growth, during which the embryonic tissue and organ systems are established; and (3) embryo maturation, during which cell expansion and/or accumulation of storage macromolecules occurs, depending on the species (Figure 1A). After embryo maturation, the embryo will lose water to enter a period of either quiescence or dormancy, depending on the species and environmental factors.

Embryogenesis in angiosperms and gymnosperm differs mainly in the sequence of events. Angiosperms cell wall synthesis begins at the first division of the zygote (Figure 1A(a,b)) whereas gymnosperms generally have a free-nuclear phase (Figure 1A(c), [6]). Among gymnosperms, it is common to see the generation of multiple embryos, which can occur via cleavage of the fertilized egg, as in conifers, fertilization of several archegonia (known as simple polyembryony) as in some cycads and taxales, or by rosette cell development (rosette polyembryony) in a few species of *Pinus* (Figure 1A(c), [7]). Regardless of the mechanism generating polyembrony initially, the mature seed contains only one embryo which has survived competition with the others. In angiosperms, polyembryony is rare; it is found in citrus species, the monocots *Cymbidium bicolor*, *Erythronium americanum*, *Nymphaea advena*, and the dicot *Nicotiana rustica*.

The partitioning that occurs following proembryo formation follows a uniform pattern of division in both angiosperms and gymnosperms, though embryogenesis after the octant stage differs between dicots and monocots. The morphological designation to the successive stages in dicots is globular, heart, torpedo, and cotyledonar, whereas in monocots is globular, scutellar and coleoptilar, (Figure 1A(a,b)).

Gamete fusion is not the only mechanism that can start plant embryogenesis. In vivo, embryo development also occurs from apomictic embryos or suspensor cells, while under appropriate in vitro conditions, embryos may develop from microspores/pollen grains or from somatic cells/tissues, known as somatic embryogenesis (SE) [8,9]. Processes related to embryo initiation and the underlying molecular control are only beginning to be understood [10,11]. Depending on the nature of the explant, a SE protocol may follow one of two developmental routes—direct or indirect. In direct SE the embryos develop directly form an explant that has intrinsic embryogenic capacity. Indirect SE involves the induction of embryogenic callus (EC), callus proliferation, embryo development and maturation, and plantlet conversion (Figure 1B). Callus induction is frequently achieved in culture medium supplemented with synthetic auxins. Then, the absence of auxin usually promotes somatic embryo development. To achieve embryo maturation, the culture medium is supplemented with maturation agents such as polyethylene glycol (PEG) and abscisic acid (ABA) [11] (see “media formulation” in Table S2). The importance of SE lies in the genetic stability that regenerated plants can offer, as well as in the potential biotechnological use for clonal propagation of plants that are profitable, agronomically important or require conservation. However, whole plant regeneration is frequently hampered by unknown molecular switches that lead to asynchronous differentiation, disturbed polarity, premature germination, loss of embryogenic potential or regeneration capacity, and strong genotypic differences in regeneration efficiency [12]. As will become evident in this review, plant regeneration failure is often due to a lack of vigor in somatic embryos because an accelerated energy metabolism prevents adequate synthesis of storage proteins or triggers premature use of storage substrates. Many important crops and grasses are recalcitrant to in vitro culturing, i.e., tissue cultures experience a decline and/or loss of embryogenic competency; these include conifers, chili pepper (*Capsicum chinese* Jacq.), coconut palm (*Cocos nucifera* L.) and avocado (*Persea americana*), among others [13,14]. The fragmented knowledge about the molecular and biochemical processes governing SE significantly constraints the establishment of reliable regeneration protocols and the opportunities for genetic transformation and/or mass production of plantlets that could result.

Proteomics is a powerful tool for determining the identity, abundance, temporal variation, and post-translational modification dynamics of proteins in association with abiotic stimuli, stress responses or development stage in any biological system [18,19]. Metabolomics aims to characterize (qualitatively or quantitatively) the small molecules in a sample to determine the direct signature of the biochemical activity behind a physiological trait or change. Proteomics and metabolomics have been considered preferable to transcriptomics approaches because they generate more precise biochemical information associated with plant embryogenesis genotypes. Proteomic analytical methods include protein separation using gel electrophoresis (usually two-dimensional electrophoresis, 2DE) or the use of liquid chromatography coupled to tandem mass spectrometry (LC-MS/MS) (see column “MS identification” in Table S1). The 2DE approach is the most used separation technique in the research papers here reported, despite presenting technical limitations, including standard deviations reported in the range of 15–70%, the inability to detect proteins outside a working pH range, loss of high molecular weight proteins [20,21], and difficulties in quantifying differentially expressed proteins (DEPs) due to co-migration [22]. Metabolomics commonly employs gas and liquid chromatography coupled to mass spectrometer analyzers (GC-MS and LC-MS, respectively) depending on the chemical volatility of the distinct metabolites. Proteomics and metabolomics approaches have significantly improved due to innovations in molecule separation, mass spectrometry (MS), and bioinformatic analysis. All of this is aided by the continuous generation of genomics information.

In this review we gather the most representative proteomics and metabolomics information in angiosperms and gymnosperms, which data are available for analysis for integrative goal. We provide the general panorama of proteomic and metabolomic studies and highlight their key findings under SE and ZE. We also emphasize the main knowledge gaps that need to be filled in order to reach a broader understanding of SE and ZE. The main subject of this review is divided into three sections: early events in plant embryogenesis (early stage of ZE/induction of SE), development and maturation during SE and ZE. Additional information related to protein extraction protocols, separation method, mass spectrometry (MS) identification, number of proteins or metabolites identified, as well as the specification of the explant and media formulation for SE studies are presented in Tables S1 and S2.

## 2. Early Events in Plant Embryogenesis

### 2.1. Ontogenetic Events during Zygotic Embryogenesis (ZE)

ZE begins with the formation of the zygote following fertilization, while somatic cells acquire embryogenic competence as a result of genomic and molecular modulations that are not yet well understood [23,24]. We found only one specific report on the early stages of ZE; this was in *Acer platanoides* L. (Norway maple) [25]. In this study, a 2DE approach suggested a fine modulation of proteins related to cellular structures (UDP (uridine diphosphate) forming α-1,4-glucan-proteinsynthase, α- and β-tubulin), as well as fundamental proteins like triose phosphate isomerase and cytochrome b6-f complex iron-sulfur protein subunit. Later proteomic studies focused on ZE were conducted using a 2DE approach, allowing the identification of only a limited number of proteins (Table S1).

### 2.2. Induction of Somatic Embryogenesis (SE)

Embryogenic potential is genotype-dependent and can be obtained through direct explant tissues or undifferentiated cultures. Several proteomics and metabolomics studies have focused on profiling differences between embryogenic (EC) and non-embryogenic cultures (NEC). Their main goal was to determine molecular and biochemical events related to the acquisition of embryogenic competence and to improve the embryogenic capacity and efficiently propagate plantlets. In the following sections, we describe selected studies in dicots, monocots, and gymnosperms with available proteomic and metabolomic data.

#### 2.2.1. Dicots

Studies in cotton (*Gossypium hirsutum*) showed the importance of epigenetic changes, reactive oxygen species (ROS) homeostasis, and interplay with auxin to modulate SE formation [26,27,28]. A proteomic comparison in barrel medic (*Medicago truncatula*), highlighted the association of EC with the efficient response to in vitro stress, which is also associated with the regulation of ROS and gibberellins [29,30]. In the medicinal plant *Nothapodytes nimmonian*, the comparison of EC and NEC suggested that the acquisition of embryogenic competence is primarily connected to the ROS scavenger activity of superoxide dismutase (SOD), catalase (CAT), ascorbate peroxidase (APX), and glutathione-s-transferases (GST) [31,32].

A recent comparative study in avocado (*Persea americana* Mill.) based on proteomics with TMT-synchronous precursor selection (SPS)-MS^3^ combined with targeted and untargeted metabolomics of EC and NEC showed that the phenylpropanoid pathway was more active in EC. The authors suggested that this pathway was linked to stress tolerance responses, probably through the reinforcement of the cell wall and production of flavonoids. Furthermore, *p*-coumaric acid and *t*-ferulic acid favored the formation of globular structures in EC [33]. An untargeted metabolomic study during SE in the medicinal plant *Silybum marianum* showed the overaccumulation of cinnamic acid, kaempferol, quercetin, myricetin, linolenic acid, and 5-enolpyruvylshikimate-3-phosphate in globular somatic embryos [34]. Other compounds like sucrose, and tryptophan, serine, cysteine, and proline were also overrepresented in globular somatic embryos in *S. marianum*.

#### 2.2.2. Monocots

In most *Zea mays* genotypes, the induction of EC is currently highly inefficient, so understanding the molecular events related to gaining embryogenic potential is an essential step for establishing in vitro production and breeding programs for maize. Three consecutive proteomics studies in maize analyzed differences between EC and NEC generated from different inbred lines (H99, A19 and Y423). The first two proteomics studies were based on 2DE and suggest the specific importance of ROS homeostasis regulation by the APX in EC and the requirement of energy by the NEC [35,36]. The third proteomics iTRAQ-based comparison showed that the metabolism of pyruvate and arginine plays a key role cell division and cell differentiation of EC [37]. Previous proteomic and metabolomic studies in contrasting EC lines (18R-high EC and B73-low EC) supported the involvement of the metabolism of amino acids, auxin, cytokinin and brassinosteroids in EC induction [22]. Metabolomics studies in *Boesenbergia rotunda* and *Brachypodium distachyon* also exhibited the essential role of amino acids (glutamine, arginine, aspartic acid, asparagine, glycine, and lysine), especially phenylalanine and tryptophan, for the acquisition of embryogenic competence [38,39].

In banana (*Musa* spp.), 2DE-based comparative proteomics showed that EC were connected with the over-accumulation of ROS scavenging proteins, heat shock proteins (HSPs) and growth regulator-related proteins like indole-3-pyruvate monooxygenase and adenylate isopentenyltransferase [40]. In addition, calcium signaling and plant growth regulators (PGRs) were also related to EC and the germination of somatic embryos. The essential role of calcium and PGRs (IAA, BAP, and kinetin) were corroborated with the proper induction of five recalcitrant banana varieties [41]. In oil palm (*Elaeis guineensis,* an interspecific hybrid between *E. oleifera* × *E. guineensis*) the predominant proteins during SE induction were those associated with storage, cellular proliferation, stress response, and energy production [42,43]. A recent phosphoprotemic study showed that proteins associated with the molecular functions of protein/nucleotide/ion binding, transferase and kinase were significantly up-accumulated during EC acquisition [44].

#### 2.2.3. Gymnosperms

Fraga et al. [45] evaluated the effect of PGRs in global DNA methylation (GDM) levels in *Araucaria angustifolia*. EC with PGRs exhibited a gradual increase in GDM levels over time in long-term subcultures, which was associated with overly active polar auxin transport, a uniform pattern of cell dedifferentiation, and maintenance of the cells in an undifferentiated state. In contrast, EC without PGRs exhibited an overaccumulation of stress-related proteins. A recent study of embryogenic suspension cultures evaluated the inhibition effect of the monopolar kinase Spindle 1 (Mps1, linked to progression of the cell cycle) on the regulation of carbohydrate, nitric oxide and polyamine (PA) contents. That study found a significant inhibition of EC growth correlated with reduced contents of endogenous sucrose, NO, and spermidine. Comparative proteomics showed a down-accumulation of proteins associated with the regulation of cell division, carbohydrate metabolism, and folding, whereas proteins related to redox processes, late embryogenesis-abundant proteins (LEAs), peroxisomal NAD-malate dehydrogenase 2 and proliferating cellular nuclear antigen 1 were up-accumulated [46].

In Douglas fir (*Pseudotsuga menziesii*), there was a stronger response to SE induction of EC derived from primary (1ry) or secondary (2ry) cotyledonary somatic embryos than from zygotic embryo material. The increase in embryogenic potential was accompanied by the up-accumulation of cathepsin B-like, E3 ubiquitin protein ligase ARI1 and ABA-inducible serine carboxypeptidase as well as the histological improvement of cellular organization of EC. Increases in embryogenic potential were also related to epigenetic mechanisms due to the up-accumulation of methyltransferase DDB and ARI1 as well as the proteome shifts induced by the interaction of the repeated intake of auxin with signaling agents such as flavonoids, ABA, JA and salicylic acid, during the three cycles [47]. Taking advantage of the potential induction of 2ry embryogenic samples, the same team carried out an integrative study that included microcopy, transcriptomics, proteomics, and metabolomics. That study showed that EC exhibited an active cytokinin metabolism linked to cellular differentiation, while NEC showed a metabolism related to stress stimuli (ABA response and oxidative stress). Auxin, isoprenoids and aromatic cytokinin were proposed as markers of EC formation [48].

An untargeted metabolomics study was carried out from embryogenic cultures to somatic embryo maturation of three *Picea abies* cell lines with different somatic embryonic development and plant formation capabilities (normal, aberrant, or blocked embryo development). Embryo proliferation and differentiation stage analyses supported the essential role of auxin due to the presence of tryptophan in the two cell lines that were able to form mature somatic embryos [49]. In addition, it was possible to observe the regulation of stress response and the occurrence of stimulatory metabolites during late stages of embryo development. Previous studies strongly suggest that also in gymnosperms, proteins connected to the stress response as well as a defense mechanism, energy metabolism, and biosynthesis of cell wall components play an essential molecular role during the early stage of SE as observed in *Pinus nigra* Arn. [50].

In *Picea balfouriana,* embryogenic ability is easily lost during long-term culture. In this context, different concentrations of 6-benzylaminopurine (BAP, 2.5 μM, 3.6 μM, and 5 μM) in proliferation medium were studied using a metabolomics-based approach. Levels of asparagine, aspartate, isoleucine, and leucine were increased in tissues with higher embryogenic ability. Reduced embryogenic competence was related to the accumulation of galactose 1, xylose 1 and fructose 2. The best efficiencies were observed at lower concentrations of 6-BAP, especially during long-term culture [51].

#### 2.2.4. General Analysis

**Figure 2:**
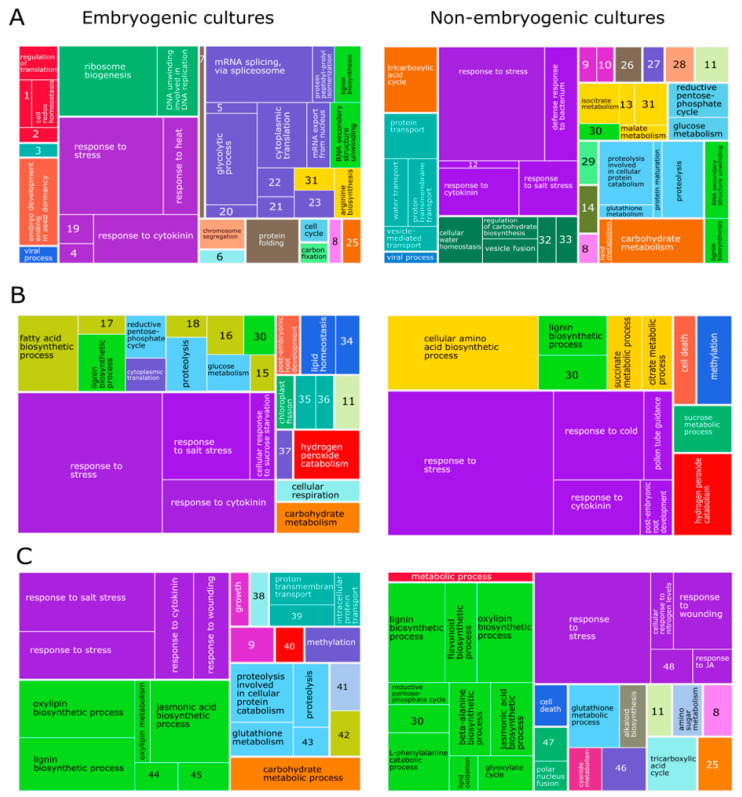
Tree Map of biological process of proteins overaccumulated in EC or NEC based on available proteomics approaches. The gene ontology enrichment of proteins was carried out with DAVID Bioinformatics Resources 6.8 and Revigo (http://revigo.irb.hr/, accessed on 27 September 2021) for reduced visual gene ontology terms. Visual representation of data related to dicots (**A**), monocots (**B**) and gymnosperms (**C**). Numbers in figure: negative regulation of long-day photoperiodism, flowering (1), positive regulation of gene expression (2), metabolic process (3), DNA ligation involved in DNA repair (4), vitamin E biosynthesis (5), microtubule-based process (6), protein refolding (7), biosynthetic process (8), unidimensional cell growth (9), seed coat development (10), photorespiration (11), pyrimidine nucleobase metabolism (13), photosynthesis (14), ‘de novo’ GDP-L-fucose biosynthetic process (15), ‘de novo’ pyrimidine nucleobase biosynthetic process (16), wax biosynthesis (17), cell wall pectin metabolism (18), response to other organism (19), amylopectin biosynthesis (20), starch biosynthesis (21), DNA replication (22), transcription by RNA polymerase I (23), chromosome segregation (24), carbohydrate metabolism (25), protein folding (26), ATP metabolic process (27), selenium compound metabolism (28), glycerol-3-phosphate metabolism (29), one-carbon metabolism (30), cellular amino acid biosynthesis (31), cell plate assembly (32), actin filament bundle assembly (33), positive regulation of RNA polymerase II transcription preinitiation complex assembly (34), protein targeting to chloroplast (35), calcium ion transmembrane transport (36), glycosyl compound metabolism (37), cortical microtubule organization (38), intra-Golgi vesicle-mediated transport (39), cell redox a homeostasis (40), amino sugar metabolism (41), dTDP-rhamnose biosynthesis (42), glycoside Catabolism (43), L-ascorbate biosynthesis (44), (R)-2-hydroxy-alpha-linolenate biosynthesis (45), mRNA splicing, via spliceosome (46), heterochromatin organization (47), heterochromatin organization (48).

**Figure 3:**
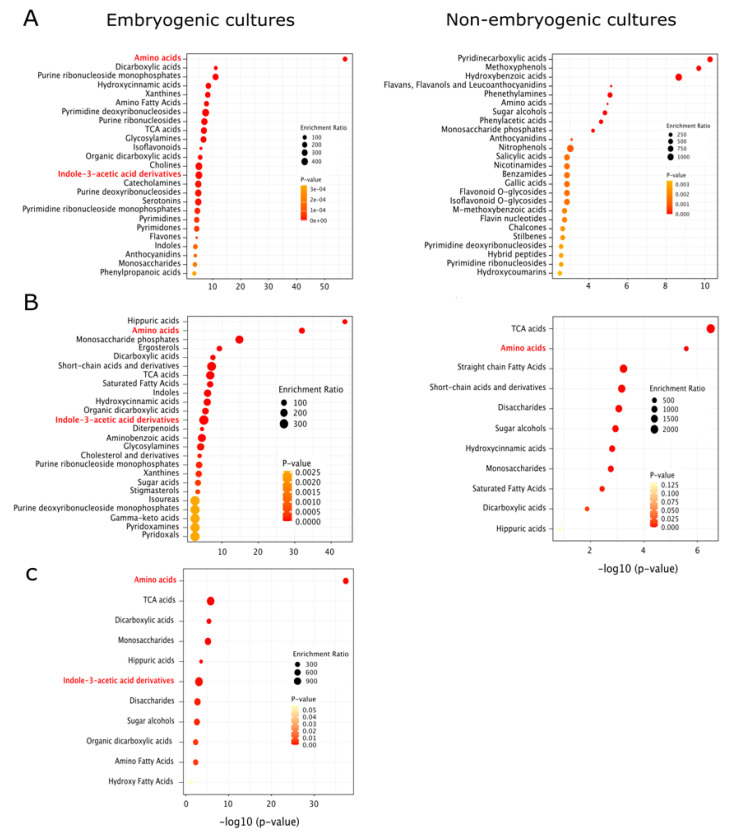
Enrichment analysis of available metabolomics data in EC and NEC in dicots (**A**), monocots (**B**) and gymnosperms (**C**). We computed KEGG annotations in metaboanalyst 5.0 bioinformatic platform (https://www.metaboanalyst.ca/, accessed on 27 September 2021). We used the library of 1072 sub chemical class metabolite sets.

**Figure 4:**
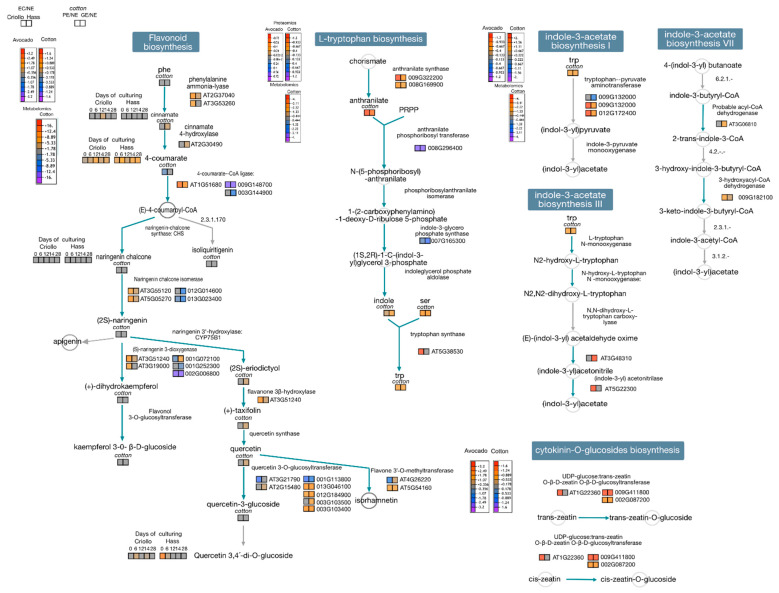
Flavonoids, L-tryptophan, indole-3-acetate and cytokinin-O-glucosides pathways-based plant metabolic pathways data based (https://plantcyc.org/, accessed on 27 September 2021). For *P. americana*, Arabidopsis homologs were used. We used fold change values and spot intensities of proteins and concentration of metabolites reported in each study. We used KEGG annotation for metabolite analysis.

Gene ontology enrichments and clustering of ontological terms showed that in dicots, monocots, and gymnosperms, proteins related to stress condition were more prominent in NEC than EC (Figure 2). However, observation highlights the occurrence of proteins related to cell redox homeostasis in EC. In general, controlled stress conditions along with a regulated oxidative status and endogenous content of growth regulator could provide the optimal biochemical and molecular situation for embryo competency. An overview of enriched metabolite sets showed contrasting pattern between EC and NEC. We highlight the over-representation of indole-3-acetic acid derivatives and amino acids in EC than NEC in dicot, monocots, and gymnosperms. In dicot EC, there is also an apparent overrepresentation of hydroxycinnamic acids, isoflavonoids, flavones, anthocyanidins and phenylpropanoic acids compare to NEC (Figure 3). Due to these observations, we focused on the biosynthetic pathways of flavonoids, L-tryptophan, indole-3-acetate and cytokinin-O-glucosides in available proteomic and metabolomic data in cotton and avocado (Figure 4), which shows contrasting data associated with flavonoid biosynthesis. In avocado EC, we could visualize a general activation of early flavonoid biosynthetic pathway compared to cotton EC. However, in cotton EC, there was an overaccumulation of (2S)-eriodictyol, (+)-taxifolin and quercetin in globular embryos. These differences could be linked to the efficiency of SE observed in cotton, contrasting with anomalies in avocado SE where EC must cope with more stress. In addition, in both EC, tryptophan, indole-3-acetate and cytokinin-O-glucoside biosynthesis is activated. Altogether, proteomics studies highlight the essential role of ROS homeostasis and modulation of growth regulators during the acquisition of embryogenic potential.

## 3. Zygotic and Somatic Embryo Development

The proteomics and metabolomics information in ZE and SE development is fragmented, likely because each plant species has different bottlenecks during development. In the case of ZE, the main constraints for research are the asynchronous development and limited amount of biological material available to compile detailed profiles of the developmental process. In the case of SE, issues included asynchronous development, malformations and deficiencies in maturation and germination of somatic embryos. Studies related to embryo development are in their infancy, and we expect key findings in the future.

### 3.1. Dicots

*Cyclamen persicum* is a well-known, economically important ornamental crop, for which SE is desirable because it would allow for vegetative propagation of parental lines and elite plants. The first study in *C. persicum* showed that torpedo somatic embryos grown under a high concentration of sucrose have a proteome profile that resembles torpedo zygotic embryos. In these experiments, higher abundance of storage proteins was determined in zygotic embryos, endosperm, and somatic embryos [52]. A later comparative proteomics study between zygotic and somatic torpedo embryos showed an over-accumulation of stress-related proteins in somatic compared to zygotic samples, including osmotin-like proteins, antioxidant 1, peroxiredoxin type II, CAT, LEA family proteins, and heat shock protein 60. Meanwhile, proteins like seed storage proteins (11S and 7S globulins), pyruvate kinase, and Em-like protein were over-accumulated in ZE samples. In addition, “small enolase” proteins (16- to 17-kDa region of 2DE gels), suggested to be storage proteins, were also overrepresented in zygotic embryos [53]. Previous studies during SE development showed that these enolases were present during all stages, but were most significantly increased in cotyledonary somatic embryos [54]. Furthermore, a proteomics study of *C. persicum* endosperm during seed development found differential accumulation of proteins related to ABA signaling, oxidative response and storage proteins, suggesting the critical participation of endosperm during zygotic embryo development [55]. Finally, Winkelmann et al. [56] carried out a metabolomic comparison between somatic and zygotic embryos in torpedo stage that also included the endosperm and testa. The metabolome-PCA analysis confirmed the similarity between zygotic embryos and endosperm tissues previously seen using a proteomics approach [53]. In the testa, polyphenolic compounds were predominant molecules, and metabolites such as proline, γ-aminobutyric acid (GABA), myo-inositol phosphate, alanine, and raffinose were significantly more abundant than other sample. On the other hand, tryptophan, galactose, adenosine, shikimate, gluconate-1,5-lactone, ethanolamine, glucose, fructose, and citric acid were significantly more abundant in SE than in ZE. Interestingly, the endosperm had higher contents of storage-related metabolites such as xyloglucans, sucrose, arginine, aspartate, alanine, glutamate, serine, palmitic and stearic acids, and raffinose compared to zygotic embryos [56].

To better understand somatic development in cotton, a proteomics analysis used isobaric tags for relative and absolute quantification (iTRAQ) of globular and cotyledonary somatic embryos. Proteomics data showed that development was associated with proteins related to stress response, such as ROS, HSPs, and LEAs, as well as PGRs homeostasis (ABA and jasmonates). Somatic embryo development was also defined by processes involved in cell wall metabolism, lipid metabolism, respiration, and photosynthesis. In cotyledonary embryos, energy homeostasis was observed with the up-accumulation of photosynthetic proteins and the down-accumulation of proteins related to respiration and carbohydrate metabolism. Proteomic results were confirmed with exogenous treatment with ABA and jasmonic acid (JA), specially at low ABA concentration (<0.04 μM) which also increased the number of secondary somatic embryos, probably as an adaptative response to osmotic conditions [57].

Cacao (*Theobroma cacao*) is another economically important species in which SE propagation has encountered low plantlet conversion ability. A comparative proteomics study of zygotic and somatic embryos at the torpedo stage [58] showed that glycolytic enzymes were overaccumulated in SE while proteins related to TCA cycle, glyoxylate cycle, pyruvate metabolism, and ascorbate metabolism were overrepresented in ZE. The authors suggested that early accumulation of aspartic protease (involved in storage protein catalysis) in zygotic torpedo embryos might be a marker of maturation onset, since it did not occur in SE.

### 3.2. Monocots

Comparative proteomics in rice using iTRAQ at different stages of ZE showed the importance of stress regulation during development. HSPs and ROS scavengers (peroxidase (POX), peroxiredoxins and a SOD) were differentially accumulated during ZE. Different lipid transfer proteins (LTPs) also displayed different abundances during development; at the beginning of morphogenesis, 90% of the LTPs were in low abundance, but by the middle of this stage, LTP163 reached its highest abundance, and during late embryogenesis, LTP24 was the highest [59]. In Date palm (*Phoenix* *dactylifera*) ZE at early stages, starch storage and glutelin accumulation, a fermentative metabolism, high levels of HSP70, and the presence of 17.6 kDa HSP were observed. Malate dehydrogenase was highly accumulated at the germination stage assayed, and UTP-GPUT (UTP-glucose-1-phosphate, uridylyltransferase) was constant throughout development, which probably indicates a permanent carbohydrate (sucrose/starch) metabolism. Furthermore, in oil palm (*Elaeis guineensis*), phosphoproteomic analysis showed that proteins associated with the molecular functions of protein/nucleotide/ion binding, transferase and kinase were identified during EC acquisition, somatic embryo development and plantlet regeneration, while pollen-specific LRR extensin-like protein 2 was specifically expressed from the callus to the cotyledonary stage [44].

### 3.3. Gymnosperms

Two consecutive proteomics studies in *Araucaria angustifolia* ZE (zygotic embryonic axis tissues of the torpedo, pre-cotyledonary, cotyledonary, and mature stages) showed the possible implication of ABA during SE development as well was the implication of proteins related to redox homeostasis and auxin metabolism, such as the APX and patellin-3. In addition, mature tissues showed overrepresentation of proteins like vicilin-like proteins and enzymes involved in glycolysis and the TCA cycle [60,61]. Shi et al. [62] and Zhen et al. [63] characterized *Cunninghamia lanceolate* ZE using 2DE at the polyembryony, dominant embryo, columnar embryo, and early cotyledonal stages. Shi et al. [62] proposed the presence of PCD proteins as a mechanistic link with embryo patterning, supporting previous studies in *P. abies* [64]. In the precotyledonary stage, Zhen et al. [63] identified the higher expression of two legumin-like storage proteins, a LEA, ROS scavengers, and a small HSP, as well as the accumulation of a “maternal effect embryo arrest” protein. They reported a low number of identified proteins since they used the EST database available for Pinaceae.

In *Pinus pinaster*, reduced water availability favored somatic embryo development. Transcriptomics, proteomics, and targeted metabolomics studies of embryo development in the presence of gellan gum showed that overproduction of ABA during development was associated with the activation of ABA signal pathways, the ubiquitin/proteasome pathway, flavonoid pathways, ROS-scavenger proteins, proteins involved in cell division, embryogenesis, and starch synthesis [65].

### 3.4. General Analysis

The limited proteomics and metabolomic studies during embryo development in plants only allowed us to gather information related to dicots. Those analyses showed differences between somatic and zygotic embryos at the torpedo stage. Proteins related to embryo maturation, glyoxylate cycle, regulation of photorespiration, regulation of fatty acid beta-oxidation, serin biosynthesis, and malate metabolisms were overrepresented in zygotic embryos (Figure S1). In somatic embryos, in addition to the torpedo stage, major biological processes at the globular stage were related to stress response and sugar metabolism, and proteins associated with photosynthesis and stress response were overrepresented in cotyledonary somatic embryos. Our analysis highlights the stress condition governing SE and the requirement of energy for the generation of plantlets. Zygotic embryos have enough building blocks such as amino acids, disaccharides, pyrimidines, and amino fatty acids, compared to somatic embryos. SE metabolic analysis showed the over-representation of 1,2-amino alcohols, gallic acids, and cyclitols, which suggest a predominant stress condition.

## 4. Zygotic and Somatic Embryo Maturation

The maturation stage of ZE is generally associated with storage reserve accumulation and physical drying. Storage reserves will determine physical drying and the subsequent acquisition of desiccation tolerance, dormancy preparation, and germination program. Even though there is a reduction of metabolic activity compared to earlier stages, maturation is an energy-demanding process that is limited by the zygotic embryo’s decreased access to oxygen. In contrast, somatic embryos continue to grow and differentiate, without a quiescent stage (as occurs in recalcitrant seeds). Even though they do not desiccate and become dormant, somatic embryos also express genes that are ABA-inducible and generally associated with desiccation tolerance in the zygotic system [66]. Acquisition of desiccation tolerance appears to be part of normal embryo development in that dehydration causes the physiological switch from favoring embryo development to processes that lead to plant development, with the catabolism of storage material upon rehydration [67]. The vast majority of studies on SE (Table S1) has focused on the maturation stage, since low conversion ability is a recurrent conundrum in somatic culture. It is generally accepted that germination potential and plantlet vigor is dependent on the accumulation of storage compounds, the readiness to release nitrogen for growth, and sugars and lipids as energy suppliers until the launch of the photosynthetic apparatus. Non-optimal germination of somatic embryos may be due to the shortage of these developmental responses determined by in vitro conditions. Constraints on plantlet conversion rates have been tackled by modifying the maturation medium to assess changes in lipid, starch, and protein content.

### 4.1. Dicots

The plant species analyzed in this section include important oil producers such as pennycress (*Thlaspi arvense* L.) and *Jatropha curcas*. The main goal of ZE in these species was to understand the biochemistry of seed development to improve the storage of high-value compounds through breeding or biotechnology techniques. Proteomics and metabolomics tools showed that sugar (sucrose, glycolysis, and photosynthesis), fatty acid (erucic acid), and amino acid metabolism (alanine, asparagine, aspartate, glutamate, glutamine, proline, and serine) plays a key role during embryo maturation [68,69]. Norway maple (*Acer platanoides* L.) is a common tree with multiple uses; a proteomics study durign ZE maturation showed that proteins like glutathione S-transferase, monodehydroascorbate reductase (MDAR), APX, LEA D-34, and dehydrins were associate with desiccation tolerance, which could provide protection during deep embryo-based dormancy [25]. Proteins like glycine-rich RNA-binding proteins, proteasome proteins, aldolase, fructose-bisphosphate aldolase, and enolase were overaccumualted in ZE maturation of Norway maple. In addition, edible plant products like *T. cacao* and *Carica papaya* were important subjects of research to improve somatic embryo maturation. In *T. cacao*, a comparison between SE and ZE showed that stress-related proteins like aspartic proteinase, PR-protein P2, and heat shock cognate 70 kDa protein were commonly overaccumulated during the maturation stage of SE and ZE. Using osmotic agents like sucrose to improve SE maturation, it was possible to visualize the accumulation of higher amounts of storage proteins and other stress-related proteins such as LEA D19 and nucleoside diphosphate kinase, during SE maturation [70]. In addition to sucrose, ABA has been used to improve the SE maturation stage of *C. persicum* Mill. [54]. In this study, stress related proteins were up-accumulated while proteins related to primary metabolism were down-accumulated under ABA and sucrose treatments. Proteins related to maturation processes, such as 7S globulin, small enolases, HSP70, and auxin-amidohydrolase were more abundant in 28 day-old embryos exposed to a higher sucrose level. In a study of *C. papaya* with a similar goal of improving the maturation of SE, EC was evaluated under light-emitting diode (LED) and fluorescent lamps. The overaccumulation of proteins associated with auxin metabolism and transport, energy production, cell wall remodeling and transport were linked to the improvement of somatic embryo maturation [71].

### 4.2. Monocots

The comparison of mature somatic and zygotic embryos of Date palm (*P. dactylifera*) showed contrasting proteomic profiles. The major observation was the identification of more stress related proteins in zygotic embryos compared to somatic embryos. It was also possible to identify HSPs and glutelin in ZE, along with proteins belonging to the glycolytic pathway, citrate cycle and ATP synthesis [72,73]. The lower accumulation of storage proteins in SE suggests a way in which culture media could be improved. This former constrains were approached by testing different concentrations of sucrose and ABA in maintenance medium for the subculture of globular somatic embryos [74]; this improvement to the medium led to a decrease in the active metabolism of SE and induced the activation of storage and defense related proteins, which made it more similar to the zygotic embryo. In sugarcane *(Saccharum officinarum*), a similar approach with the addition of activated charcoal improved the maturation process of EC. Proteins related to stress were overaccumulated in EC, such as the putative drought-inducible protein 1OS, desiccation-related protein pcc13-62, callose synthase 1, and nitric oxide synthase. A similar pattern was observed for proteins related to aromatic amino acid metabolism, like 3-deoxy-d-arabino heptulosonate-7-phosphate synthase, and anthranilate phosphoribosyltransferase, which is associated with the biosynthesis of tryptophan. At the end of the maturation process, germin-like protein, embryo-specific protein, and kinase interacting protein 1 (CIPK) were up-accumulated compared to day 0 [75]. A later study focused on the effect of PA on somatic embryo maturation by the proteomic comparison of EC and NEC cultured in somatic embryo induction medium; it showed that 500 μM putrescine induced changes in the accumulation dynamics of arabinogalactan proteins (AGPs), HSPs, POX, GSTs, 14-3-3, LEA and ubiquitin-like protein. The differential accumulation of these proteins suggested that higher exogenous putrescine concentrations provided a protective mechanism against the oxidative stress of in vitro conditions, leading to the formation of more somatic embryos [76]. Proteomic studies on wheat (*Triticum aestivum*) zygotic embryos based on 2DE showed the importance of starch, storage proteins and aminoacidic metabolism at the beginning of the maturation process, while in the embryo it was possible to identify stress-related proteins like HSP70. At the last stage of maturation, LEA proteins were the prominent protein in zygotic embryos [77].

### 4.3. Gymnosperms

The first study was a comparative study in *Araucaria angustifolia* conducted by 2DE and label free between mature and germinated embryos from seed. The analysis showed that mature tissues were associated with the overaccumulation of the storage proteins vicilin-like protein and carbamoyl phosphate synthase compared to germinated samples. [78]. Embryo maturation and germination is essential to understand ZE and extrapolating this information to SE. In this context, Jo et al. [79] carried out in *A. angustifolia* SE a proteomic screening of nine EC in the proliferative stage to determine their ability to develop somatic embryos in response to specific maturation conditions. They found marked differences between cell lines generated from cotyledonary somatic embryos. SE that were responsive to maturation conditions had an over-accumulation of S-adenosylmethionine (SAM) synthase, mitochondrial ATPase beta subunit and a probable elongation factor II. ATPase was suggested as the marker for the selection of EC lines responsive to maturation treatments.

As part of this effort in *Pinus pinaster*, a comparison of 2DE-based proteome profiles of cotyledonary (10 or 12 weeks of maturation) somatic and zygotic embryos showed a high level of similarity (94%) in carbohydrate and protein content, represented by HSPs, LEA, vicilin-like storage protein, adenosine kinase 2, and SAM synthase. However, storage proteins and cupin-related proteins were present in higher proportion in ZE than in SE, indicating their key role in tolerance to desiccation during plant embryogenesis [80]. Although SE in *P. pinaster* is well characterized, some pitfalls are associated with the transition of mature somatic embryos into whole plants, causing reduced yields. Consequently, these results could provide molecular markers of embryo quality with respect to storage compounds, since the morphologically guided determination of maturation state of somatic embryos can be misleading. In the same vein, the effect of carbohydrates and osmotic balance in the development, maturation, and germination was evaluated in *Picea abies* with the aid of targeted metabolomic and proteomic analyses. The high levels of sucrose, raffinose and LEA proteins in embryos treated with 3% sucrose were associated with the improvement of somatic embryo germination, probably by promoting desiccation tolerance [81]. Later, proteomics studies during partial desiccation of *Picea asperata* somatic embryos showed that stress-related proteins associated with osmosis, PGR metabolism, antioxidative proteins, defense proteins, and photosynthesis-related proteins were critical factors for improving the maturation and germination of somatic embryos [82]. Besides, high-temperature application during the initiation of embryonal masses of Pinus radiata exhibited improvement in the generation of the number of somatic embryos, which was associated with the over-accumulation of stress-related proteins and enzymes connected to the synthesis of fatty acids, myo-inositol, and cell-wall [83].

In *Pecea glauca* a metabolomic analysis based on nuclear magnetic resonance (NMR) was carried out in somatic cell cultures, either in maintenance medium or maturation medium, to determine which changes led to a fully developed somatic embryo [84]. The difference between the maintenance and maturation stage was the production of valine, phenylalanine, glutamine in the former, and short chain keto-acids (SCKAs) and branched chain keto-acids (BCKAs) in the latter. These amino acid metabolites may act as building blocks for proteins and polyphenolics that are indispensable for the maintenance of somatic embryos in culture. In contrast, BCKAs and SCKAs dominated the metabolic footprint of the maturation medium, which could alter CoA biosynthesis.

### 4.4. General Analysis

**Figure 5:**
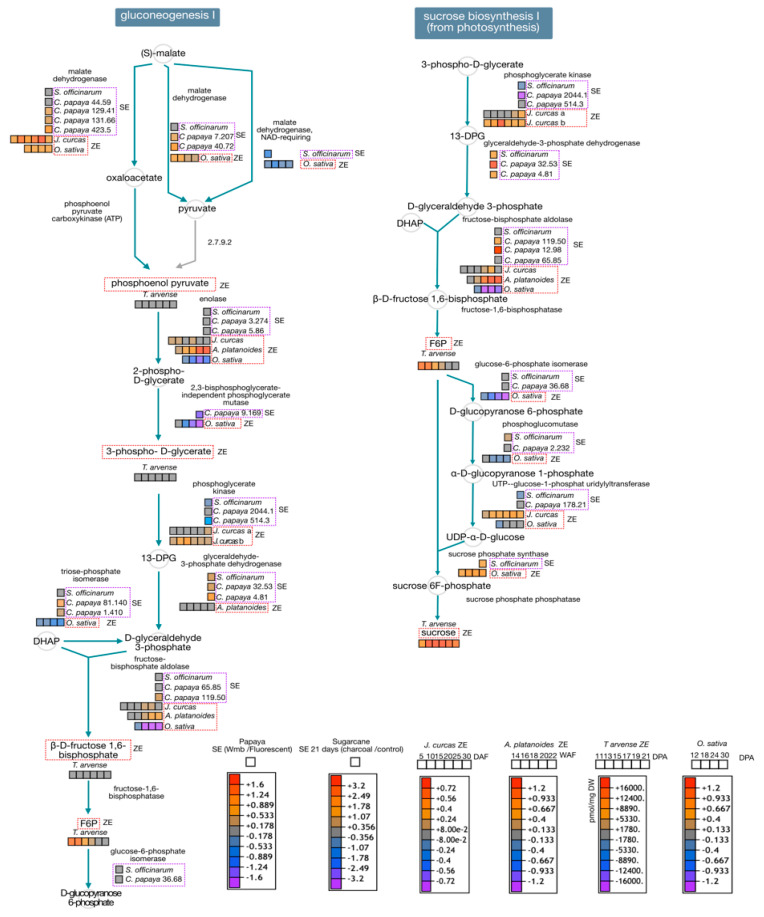
Visual representation of gluconeogenesis, and sucrose biosynthetic metabolic pathways highlighting the maturation stage of somatic and zygotic embryos. We used Arabidopsis homolog proteins for *J. curcas*, *A. platanoides, S. officinarum* and *O. sativa*. We built the metabolic pathways as indicated in Figure 4.

Proteomics and metabolomics studies pointed out that sugar metabolism during embryo maturation is essential to store energy for future germination. By analyzing available data, we take a closer look at gluconeogenesis and sucrose biosynthesis (Figure 5). We include proteomics data associated with the improvement of maturation of somatic embryos of *C. papaya* by LED and fluorescent lamps, maturation of zygotic embryos of *J. curcas* and *A. platanoides*, SE of *S. officinarum* treated with charcoal and ZE of *O. sativa*, as well as metabolic information of *T. arvense* ZE. We could suggest that those treatments with LED and fluorescent lamps improved the maturation of somatic embryos probably by activating the gluconeogenesis, and sucrose metabolic pathways. Malate dehydrogenase and fructose-bisphosphate aldolase were over-accumulated during somatic and zygotic embryo maturation, while enolase, phosphoglycerate kinase and fructose 6-phosphate (F6P) were over-accumulated in zygotic embryos. Sucrose was overproduced during zygotic embryo maturation of *T. arvense,* highlighting the essential role of this polysaccharide in embryo maturation. Phosphoglycerate kinase, and UTP-glucose-1-phosphate uridylyltransferase were over-accumulated in ZE, while glyceraldehyde-3-phosphate dehydrogenase was over-accumulated SE and fructose-bisphosphate aldolase were over-accumulated in SE and ZE. In addition, proteomics publications in dicots, monocot and gymnosperms highlight the importance of storage proteins like globulins, vicilin and small enolases during embryo maturation, as well as the overrepresentation of stress related proteins like LEA and other desiccation-related proteins.

## 5. Perspectives: What We Know about ZE and SE

**Figure 6:**
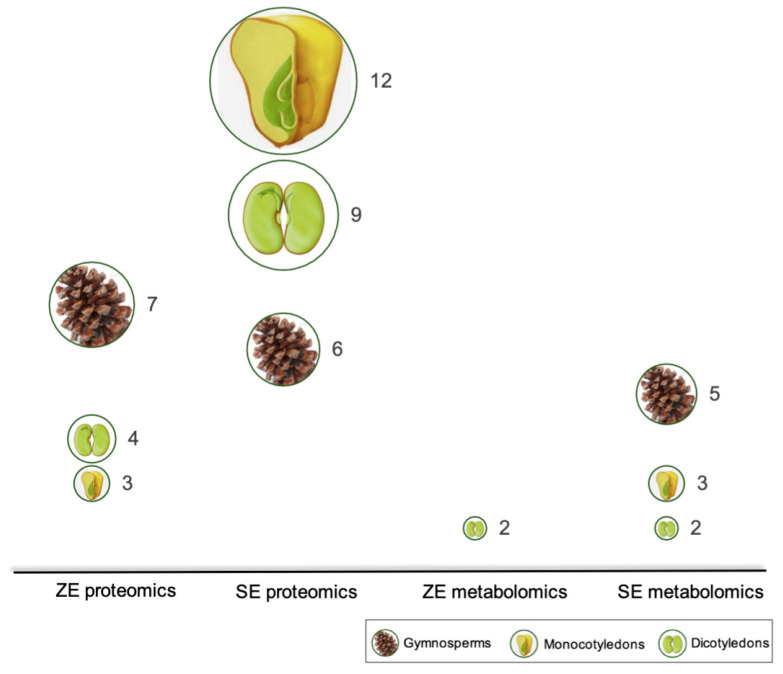
Number of publications on zygotic and somatic embryogenesis in angiosperms and gymnosperms using proteomic and/or metabolomic approaches in the last decade. Bubble and illustration size are proportional to the number of reviewed publications and plant group, respectively.

A first glance at proteomic and metabolomics studies in plant embryogenesis shows an imbalance in the number of publications among dicots, monocots, and gymnosperms (Figure 6). We found more proteomics studies in SE than ZE, and monocots have the highest number of publications, followed by dicots and gymnosperms. The fragmented information in proteomics and metabolomic studies in plant embryogenesis is an obstacle to integrating information and drawing conclusions. A significant number of proteomics and metabolomics studies addresses events during late maturation, such as desiccation, dormancy, and germination, which are beyond the scope of this review. Nevertheless, we could identify general differences and opportunity areas for future research by dividing our review into the three main stages of development: early embryogenesis (induction of SE or early events of ZE), development, and maturation.

### 5.1. Induction

Comparison between EC and NEC was the richest area of research in our review. EC induction depends on the genotype, source of explant, and the concentration of PGRs in the culture medium. During EC acquisition and induction, nitrogen metabolism, stress, and defense proteins to control oxidative stress, proteins related to meristem initiation and cell cycle regulation were the most reported processes. EC proliferation was also characterized mainly by the abundance of protein recycling and folding, and proteasomal degradation-related proteins. Epigenetic modifications during EC establishment were reported in some publications, but this subject deserves another review. In contrast, NEC was characterized by the overabundance of flavonoids, anthocyanidins and anthocyanins, inducing unpolarized or irregular structures, as well as lower abundance of proteins and enzymes involved in DNA repair. Metabolomics data reinforced the proteomics knowledge of EC induction; tryptophan is the precursor of key PGRs, glutamine is a nitrogen source, arginine is a precursor for PA biosynthesis, and phenylalanine is precursor of polyphenolic compounds. Our general analysis showed that the fine regulation of the tolerance of stress response, ROS-homeostasis and content of PGR play fundamental roles during the generation of EC.

The first evident need is to increase the number of studies of early ZE to balance with the number of SE publications. In this context, it is important to mention that there is evidence in maize, rice and *Arabidopsis* that gamete-proteins may play a role in determining cell fate during early embryogenesis. A 2DE based comparison (without LC-MS/MS analysis) of protein content from maize egg-cells and two-celled embryos showed differences between their silver-stained band intensity [85]; a later single-cell proteomics comparison of rice gametes showed egg- and sperm-specific proteins that had not been previously related to reproduction or developmental processes [86]. Therefore, we emphasize the need to carry out more studies on ZE, although the endeavor of sampling manageable quantities of embryonic tissue during early ZE, or its asynchrony in nature are significant methodological hurdles to understanding the biochemistry of cell-fate determination by asymmetrical division. Although it is generally accepted that the resemblance of the somatic embryo to the zygotic embryo will give rise to satisfactory outcomes in in vitro plant culture, the number of publications related to ZE omics still does not provide enough information to be used as molecular tool for improving SE (Figure 6).

We suggest the application of novel tools to overcome factors like the complex chemistry of plant tissue, dynamic range of proteins (lower or higher abundance of proteins and metabolites), and small amounts of biological materials to sample, which make it difficult to make detailed characterizations of the proteome and metabolome. These tools include single-cell-type proteomics and/or the miniaturization of proteomics pipelines. This latter approach may involve protoplast isolation, capillary micropipetting, cell sorting, laser capture microdissection [87], or the fractionation of cell organelles with Percoll, INTACT (Isolation of Nuclei Tagged in specific Cell Types), iodixanol or sucrose-gradient [88]. Although these isolation methods modify the molecular condition of the cell and tissues, they provide critical information regarding the molecular mechanisms behind biological processes. Furthermore, targeted metabolite profiling represents a powerful tool to monitor the changes and delicate balance of PGRs such as auxin, ABA, JA, ethylene, and PA. Untargeted metabolomics bias identification towards abundant primary and some secondary metabolites and exclude most low-abundance compounds such as PGRs, even when using high-resolution mass spectrometry. Validation of the identity of putative metabolites against commercial standards is a prerequisite; this validation, along with robust metabolomic studies, will serve to elucidate the role of these molecules and other metabolites in plant embryogenesis.

### 5.2. Development

The histological and morphological characterization of somatic and zygotic embryos of some plant species have allowed the identification of specific and general features of plant SE. However, to date there has been no detailed characterization of the temporal and spatial patterns of ZE or SE proteomic and metabolomic profiles. Based on proteomics data, we know that the signature processes of early development are stress responses, ROS signaling and scavenging, energy metabolism, growth, cell cycle regulation, and cell differentiation. Metabolomics information reveals the active production of amino acids, organic acids, and secondary metabolites. We also observed that embryo transdifferentiation during development is characterized by proteins related to cytoskeletal organization, cell wall remodeling, auxin homeostasis and polar distribution, and flavonoid biosynthesis (Figure S2). Studies in dicots provide some information that allows us to compare the torpedo stage of SE and ZE. Stress-related proteins are overrepresented in SE compared to ZE, while proteins related to photosynthesis are found exclusively in ZE, which was associated with higher disaccharide content. These results show the scattered nature of the information on embryo development, which prevents the use of information on ZE to improve SE. Proteometabolomic information, together with statistical approaches, will make SE culture improvement a rational procedure rather than an empirical one in which efforts are mostly unguided, and inconsistencies in the results may be mistakenly attributed to biological variability. This reinforces the observation by many studies that SE morphology cannot be used to guide EC selection.

### 5.3. Maturation 

During the maturation stage, a consistently reported difference between ZE and SE is the higher abundance and specialization of storage compounds in zygotic embryos (e.g., glutelins, small HSPs, LEAs, dehydrins, raffinose, among others). Different treatments, such as sucrose, ABA, activated charcoal and other stressors have been used to induce maturation and germination of somatic embryos. Some studies of the proteome profile showed the induction of the above-mentioned storage- and stress-related proteins in somatic embryos under stress. Nevertheless, several pitfalls during the maturation and germination stages are related to molecular alteration at the induction stage of EC. Therefore, detailed proteomics and metabolomics studies in ZE of each plant species, as well as plants species with well-stablish SE processes, should be considered as proper controls. Finally, it should be noted that the small number of identified proteins (Table S1) reflects the need to enlarge the genome-transcriptome databases of orphan or non-model organisms that are currently absent from public databases.

## 6. Conclusions

Current proteomics and metabolomics information suggests key roles of stress, ROS homeostasis, and endogenous PGR content during the early and developmental stages of plant embryogenesis (ZE and SE). However, it is not yet clear how stress-induced dedifferentiation leads to cellular totipotency only in certain cells of certain genotypes. During the maturation stage, there is an over-accumulation of storage proteins for future germination and biochemical preparation to cope the desiccation period. However, the fundamental biochemical knowledge of plant cellular totipotency is still a mystery. Our review shows the fragmented body of knowledge of the proteometabolome of plant embryogenesis, in which research has targeted different developmental stages, different culture conditions, and different objectives-based requirements for improving somatic embryogenesis. However, most of the pitfalls of SE are specific to the genotype of each plant species. Therefore, a complete proteomics and metabolomic characterization of each stage of ZE will provide solid hypotheses to corroborate the success of SE. We predict that in the future, the use of more robust proteomics and metabolomics tools. Implementation of new protocols will help fill in the current gaps in proteomic and metabolomic information on ZE.

## Data Availability

Not applicable.

## Supplementary Materials

Supplementary materials can be found at https://www.mdpi.com/article/10.3390/ijms222111807/s1. Figure S1: Visual representation of biological processes of identified differential proteins and metabolite-based Arabidopsis homologs of studies associated with the development of somatic and zygotic embryos. The gene ontology (GO) enrichment and reduced visual GO terms was carried out with DAVID Bioinformatics Resources 6.8 and Revigo (http://revigo.irb.hr/, accessed on 27 September 2021). For metabolite enrichment analysis, we used the KEGG annotation and bioinformatic tool MetabolAnalyst 5.0, Figure S2: Functional classification of proteins and compounds highlighted in the publications here reviewed using UniProt annotation. We show the functional categories according to the analyses reported for the ontogeny, development, and maturation stages. In our analysis, we covered angiosperms (monocot and dicots) and gymnosperms. Proteomics data are in bold and metabolomics data in italics. To distinguish data reported for angiosperms and gymnosperms, text in red represents monocots, blue represents dicots, orange represents gymnosperms, and purple represents data reported in both angiosperms and gymnosperms, Table S1: Relevant data about proteomics and metabolomics regarding ZE and SE research reported in the reviewed literature (To see further information about SE experiments such as type of explant, medium formulation and growth conditions go Table S2), Supplementary Table S2: Experimental data reported in the papers reviewed about somatic embryogenesis.

## Abbreviations

ABA	Abscisic acid
APX	Ascorbate peroxidase
CAT	Catalase
2DE	Two-dimensional electrophoresis
EC	Embryogenic callus
EMSN	Extracellular matrix surface Network
EP	Embryogenic potency
GST	Glutathione-s-transferases
HSPs	Heat shock proteins
JA	Jasmonic acid
LEAs	Late embryogenesis-abundant proteins
MS	Mass spectrometry
NEC	Non-embryogenic cultures
PGRs	Plant growth regulators
PCA	Principal component analysis
POX	Peroxidase
PP	Phenylpropanoid pathway
PA	Polyamine
ROS	Reactive oxygen species
SAM	S-adenosylmethionine
SE	Somatic embryogenesis
SOD	Superoxide dismutase
TMT	Tandem mass tags
ZE	Zygotic embryogenesis.
