# Supplementary material for: Current Proteomic and Metabolomic Knowledge of Zygotic and Somatic Embryogenesis in Plants
Source: Int J Mol Sci. 2021 Oct 30;22(21):11807. doi: 10.3390/ijms222111807 (PMC8583726; doi:10.3390/ijms222111807)
Supplement: Supplementary file 1 [file ijms-22-11807-s001.zip › Supplementary Figures.pdf]

Supplementary Figure S1. Visual representation of biological processes of identified differential proteins and metabolite-based Arabidopsis homologs of studies associated with the development of somatic and zygotic embryos. The gene ontology (GO) enrichment and reduced visual GO terms was carried out with DAVID Bioinformatics Resources 6.8 and Revigo (http://revigo.irb.hr/). For metabolite enrichment analysis, we used the KEGG annotation and bioinformatic tool MetabolAnalyst 5.0

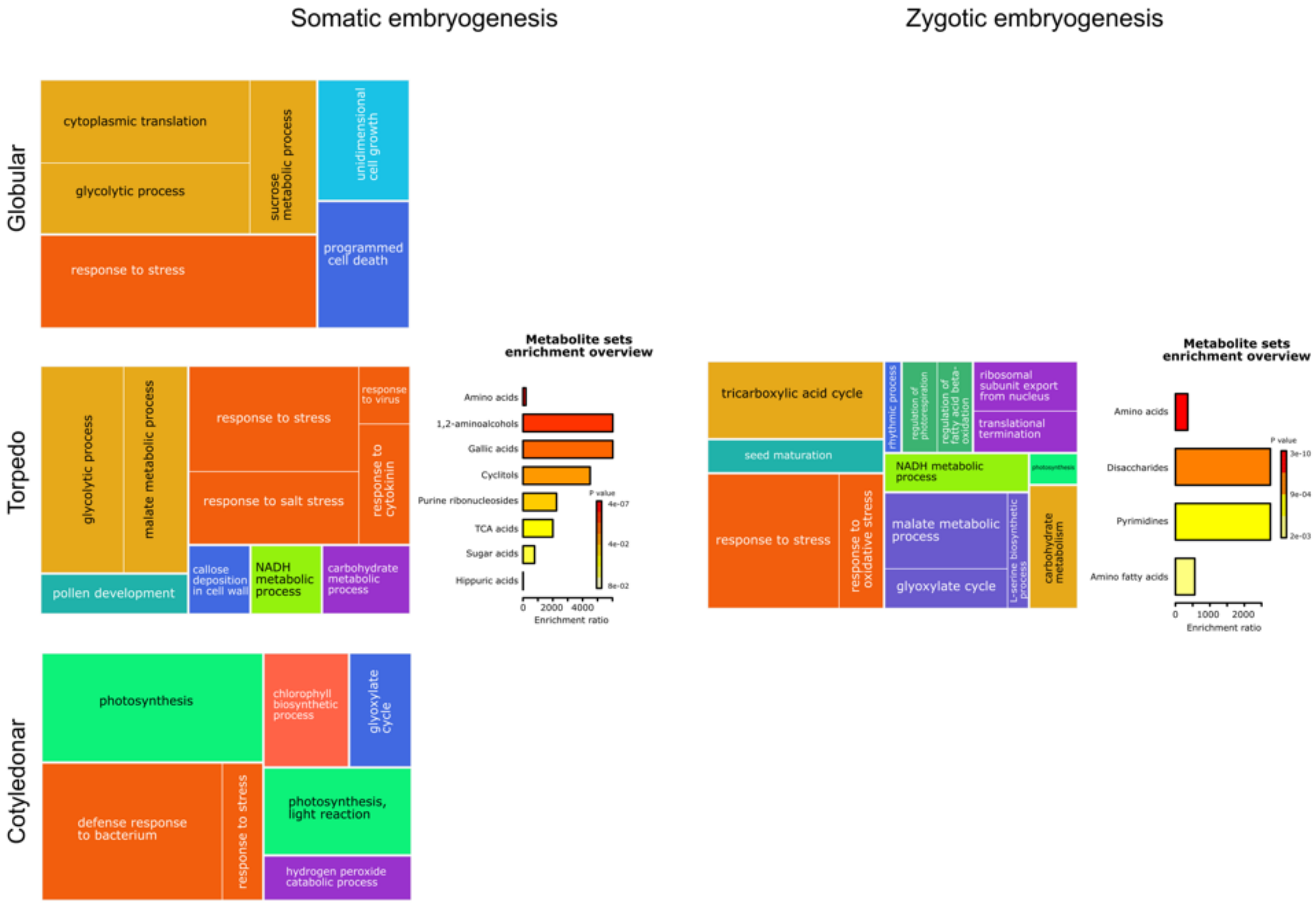

Supplementary Figure S2. Functional classification of proteins and compounds highlighted in the publications here reviewed using UniProt annotation. We show the

functional categories according to the analyses reported for the ontogeny, development, and maturation stages. In our analysis, we covered angiosperms (monocot and dicots) and gymnosperms. Proteomics data are in bold and metabolomics data in italics. To distinguish data reported for angiosperms and gymnosperms, text in red represents monocots, blue represents dicots, orange represents gymnosperms, and purple represents data reported in both angiosperms and gymnosperms.

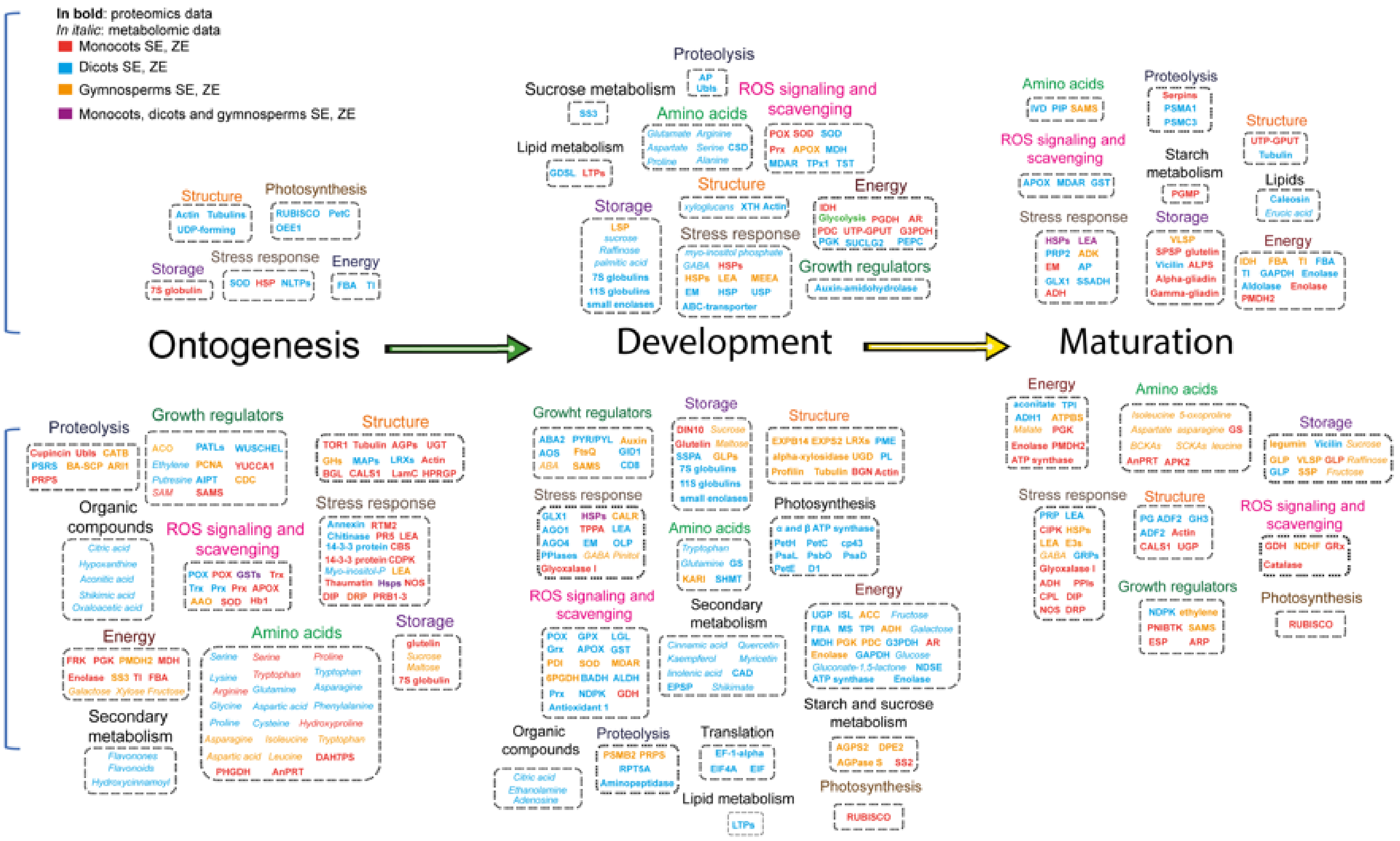

Abbreviation of proteins presented in Figure S2.

ABA receptor (PYR/PYL), Acetyl-CoA carboxylase (ACC), adenosine kinase 2 (ADK), adenylate isopentenyltransferase (AIPT), adenylyl-sulfate kinase2 (APK2), alcohol

dehydrogenase (ADH), aldehyde dehydrogenase (ALDH), aldose reductase (AR), allene oxide synthase (AOS), anthranilate phosphoribosyltransferase (AnPRT), apoplastic, anionic guaiacol peroxidase (GPX), arabinogalactan proteins (AGPs), ascorbate, peroxidase (APOX), aspartic proteinase (AP), ATP synthase CF1 alpha subunit (α) and beta subunit (β), auxin responsive (ARP), auxin-amidohydrolaseembryonic protein DC-8-like (CD8), avenin-like proteins (ALPS), 1-aminocyclopropane- 1-carboxylate oxidase (ACO), 4-aminobutyric acid (GABA), BA-inducible serine carboxypeptidase (BA-SCP), betaine aldehyde dehydrogenase (BADH), branched-chain keto acids (BCKAs), calcium-dependent protein kinase (CDPK), callose synthase 1 (CALS1), calreticulin (CALR), cell division control proteins (CDC), cell division protein (FtsQ), chloroplast stromal ascorbate peroxidase (APOX), cinnamyl alcohol dehydrogenase (CAD), class 1 hemoglobin (Hb1), cupincin-like proteases (Cupincin), cystathionine beta-synthase family protein isoform 2 (CBS), cysteine desulfurase (CSD), cytochrome b6-f complex iron-sulfur subunit (PetC), cytoskeletal organization (ADF2), D-3-phosphoglycerate dehydrogenase (PHGDH), desiccation-related protein (DRP), drought-inducible protein (DIP), 3-deoxy-d-arabino heptulosonate-7-phosphate synthase (DAH7PS), E3 ubiquitin protein ligase (ARI1), elongation factor 1-alpha (EF-1-alpha), Em-like protein (EM), embryo-specific protein (ESP), eukaryotic initiation factor 4A (EIF4A), eukaryotic translation initiation factor (EIF), expansin S2, EXPS2, 5-enolpyruvylshikimate-3-phosphate (EPSP), ferredoxin-Nadp+ reductase (PetH), fructokinase (FRK), fructose-bisphosphate aldolase (FBA), fructose-bisphosphate aldolase 2 (FBA), galactinol sucrose galactosyltransferase 6, (DIN10), GDSL esterase/lipase (GDSL), germin-like protein (GLP), glucan-endo-1,3-beta-glucosidase (BGN), glucose-1-P adenylyltransferase large subunit, (AGPase S), glucose-1-phosphate adenylyltransferase (AGPS2), glutamate dehydrogenase (GDH), glutamine synthetase (GS), glutathione S-transferase (GST), glutaredoxin (Grx), α-1,4-glucan-protein synthase (UDP-forming), β-(1,3)-glucanase (LamC), β-D-glucosidase (BGL), 4-alpha-glucanotransferase DPE2 (amylomaltase), glyceraldehyde 3-phosphate dehydrogenase (GAPDH), glycine-rich RNA-binding protein (GRPs), glycosyl hydrolase family protein (GHs), hydroxyproline-rich glycoprotein 1 (HPRGP), indole-3-pyruvate monooxygenase (YUCCA1), isocitrate dehydrogenase (IDH), isovaleryl-CoA dehydrogenase (IVD), isocitratelyase (ISL), ketol-acid reductoisomerase (KARI), kinase interacting protein 1 (CIPK), L-ascorbate oxidase homolog (AAO), lactoylglutathione lyase (GLX1), late embryogenesis abundant (LEA), legumin-like storage proteins (LSP), leucine-rich repeat extensin-like (LRXs), malate dehydrogenase (MDH), malate synthase (MS), maternal effect embryo arrest (MEEA), microtubule-associated protein (MAPs), microtubule-associated protein TORTIFOLIA1 (TOR1), mitochondrial ATPase beta subunit (ATPBS), monodehydroascorbate reductase (MDAR), NADH dehydrogenase subunit F (NDHF), NADP-dependent glyceraldehyde-3-phosphate dehydrogenase (G3PDH), nitric oxide synthase (NOS), nonspecific lipid transfer protein (NLTPs), nucleoside diphosphate kinase (NDPK), nucleoside-diphosphate-sugar epimerases

(NDSE), nucleoporin (NUP50A), osmotin-like protein (OLP), oxygen evolving enhancer protein 1 (OEE1), patellin-3-like (PATLs), pathogen-related protein(PR), pectate lyase (PL), pectin methylesterase (PME), peptidyl prolyl cis/trans isomerase (PPIases), peroxiredoxin (Prx), peroxisomal NAD-malate dehydrogenase 2 (PMDH2), phosphatase-like isoform (CPL), phosphoglucomutase (PGMP), phosphoglycerate kinase (PGK), phosphoglycerate kinase (PGK26S), photosystem I reaction center subunit II (PsaD) and subunit XI (PsaL), photosystem II 44 kDa protein (cp43), photosystem Q(B) protein (D1), plastocyanin (PetE), polygalacturunase (PG), polyphosphoinositides (PPIs), proliferating cellular nuclear antigen 1 (PCNA), proline iminopeptidase (PIP), proteasome ATPase subunit 5a (RPT5A), proteasome regulatory particle subunit (PRPS26S), proteasome subunit alpha type-1 (PSMA1), proteasome subunit beta type (PSMB2), protein argonaute 1-like isoform X2 (AGO1) and 4-like (AGO4), protein disulfide isomerase (PDI), protein RESTRICTED TEV MOVEMENT 2 (RTM2), protein/nucleotide/ion binding, transferase and kinase (PNIBTK), proteins cathepsin B-like (CATB), 26S protease regulatory subunit 6A homolog (PSMC3), 26S proteasome regulatory particle subunit (PRPS), 26S proteasome regulatory subunit (PSRS), 6-phosphogluconate dehydrogenase (PGDH), putative alpha-xylosidase, putative expansin-B14 (EXPB14), putative germin-like protein (GLPs), pyruvate decarboxylase (PDC), ribulose bisphosphate carboxylase (RUBISCO), S-adenosyl methionine (SAM) and synthase (SAMS), seed storage and (19 kDa) proteins (SSP), serine hydroxymethyltransferase (SHMT), short-chain keto acids (SCKAs), starch synthase II (SS2), seed storage protein A (SSPA), storage-protective serpin protein (SPSP), succinate malate CoA ligase (SUCLG2), succinate semialdehyde dehydrogenase (SSADH), sucrose synthase 3 (SS3), superoxide dismutase (SOD), thioredoxin (Trx), hioredoxin peroxidase 1 (TPx1), thiosulfate sulfurtransferase (TST), trehalose-phosphate phosphatase A (TPPA), triosephosphate isomerase (TI), triosephosphate isomerase (TPI), tubulin beta-2 chain, ubiquitin ligase (E3s), ubiquitin-like protein (Ubls), UDP-glucose 6-dehydrogenase (UGD), UDP-glucose pyrophosphorylase (UGP), UDP-glucuronosyltransferase (UGT), universal stress protein (USP), UTP-glucose-1-phosphate uridylyltransferase (UTP-GPUT). vicilin-like storage protein (VLSP), WUSCHEL-related homeobox 9-like protein (WUSCHEL), xanthoxin dehydrogenase (ABA2), xyloglucan endotransglycosylase (XTH).
