# Supplementary material for: Current Proteomic and Metabolomic Knowledge of Zygotic and Somatic Embryogenesis in Plants
Source: Int J Mol Sci. 2021 Oct 30;22(21):11807. doi: 10.3390/ijms222111807 (PMC8583726; doi:10.3390/ijms222111807)
Supplement: Supplementary file 1 [file ijms-22-11807-s001.zip › Supplementary Tables.pdf]

Supplementary Table 1. Relevant data about proteomics and metabolomics regarding ZE and SE research reported in the reviewed literature

(To see further information about SE experiments such as type of explant, medium formulation and growth conditions go to Supplemental Table 2)

dicots | monocots | gymnosperms

1st STAGE: Ontogeny . ZE = early stages (before globular). Direct SE= induction; indirect SE = induction and proliferation (monocots: induction to early embryogenesis (embryogenic phase); gymnosperms: EC to PEM)

Species	Sampled stage	Protein extraction	Separation	MS identication[†]	Identified elements[‡]	Type of analysis	Reference
Acer platanoides L	Morphogenesis (10 WAF)	10% (w/v) TCA in acetone containing 20 mM DTT	2DE	*(in-gel)* LC- MS/MS	17 proteins	**ZE proteomics**	Staszak and Pawłowski, 2014
Gossypium hirsutum L	NEC, PEC, GSe	NA	NA	UPLC-MS/MS	581 metabolites	SE metabolomics	Guo *et al*., 2019
Gossypium hirsutum L	NEC, PEC, GSe	Phenol-ammonium sulphate/methanol precipitation	Liquid chromatography	(TMT) LC-MS/MS	9369 proteins (6730 quantified)	SE quantitative proteomics	Guo *et al*., 2019b
Medicago truncatula	Induced and proliferating calli	Acetone	2DE	*(in-gel)* MALDI-TOF/TOF MS	136 proteins	SE proteomics	Almeida *et al*., 2012
Nothapodytes nimmoniana	NEC and EC	Phenol-ammonium acetate	2DE	*(in-gel)* MALDI-TOF/TOF MS	46 out of 97 spots	SE proteomics	Isah and Umar, 2019
Persea americana	NEC and EC	Phenol	RP chromatography	TMT-LC-(SPS)-MS3	3841 proteins	SE proteomics	Olivares-García *et al*., 2020
Brachypodium distachyon	EC	NA	NA	GC-MS	79 metabolites	SE metabolomics	Mamedes-Rodrigues *et al*., 2018
Boesenbergia rotunda	EC	NA	NA	UPLC-MS, targeted	51 metabolites	SE metabolomics	Ng *et al*., 2016
Elaeis guineensis Jacq.	explant, 14 days of induction, primary callus and pro-embryogenic callus	Phenol-0.1 M ammonium acetate in methanol	2DE	*(in-gel)* MALDI-TOF/TOF MS	45 proteins	SE proteomics	Silva *et al*., 2014
Elaeis oleifera, E. guineensis	Induction stage	Phenol	Liquid chromatography	LC-MS/MS	4695 in total; 221 daps	SE proteomics	Ribeiro *et al*., 2019
Musa spp.	EC and NEC induction	Phenol-0.1 M ammonium acetate in methanol	2DE	*(in-gel)* MALDI-TOF/TOF MS	65 proteins	SE proteomics	Kumaravel *et al.*, 2017
Zea mays	EC induction	TCA	iTRAQ-SCX	UPLC-MS and iTRAQ LC-MS/MS	314 metabolites; 4,816 proteins	SE proteomics and metabolomics	Ge *et al*., 2017
Zea mays	EC and NEC	Lysis buffer	iTRAQ-SCX	iTRAQ-LC-MS/MS	5592 proteins; 632 daps	SE proteomics	Liu *et al*., 2018
Zea mays	EC and NEC	TCA-acetone	2DE	*(in-gel)* MALDI-TOF/TOF MS	29 proteins out of 42 spots	SE proteomics	Sun *et al*., 2013
Zea mays	EC and NEC	Phenol-0.1 M ammonium acetate in methanol	2DE	*(in-gel)* Q-TOF-MS	23 proteins out of 361 spots	SE proteomics	Varhaníková *et al.*, 2014
Cunninghamia lanceolata	Early embryogeny	Lysis buffer	2DE-DIGE	*(in-gel)* LC-MALDI-TOF MS/MS	71 proteins, 136 spots	**ZE proteomics**	Shi *et al*., 2010
Cunninghamia lanceolata	Early embryogeny	Lysis buffer	2DE-DIGE	*(in-gel)* LC-MS/MS	11 proteins, 153 spots	**ZE proteomics**	Zhen *et al*., 2010
Araucaria angustifolia	EC	Acetone-chloroform	2DE	*(in-gel)* MALDI-TOF /TOF MS	11 deps	SE proteomics	Jo *et al*., 2014
Araucaria angustifolia	EC	Methanol–chloroform	2DE	*(in-gel)* LC-MS/MS	1932 (576 were annotated with Pinidae subclass data)	SE quantitative proteomics	Fraga *et al*., 2016
Araucaria angustifolia	EC	Methanol–chloroform	Liquid chromatography	LC-MS/MS	1518	SE quantitative proteomics	Douétts-Peres *et al*., 2019
Picea balfouriana	EC	NA	NA	GC/TOF/MS	123 metabolites	SE metabolomics	Li *et al*., 2015
Pseudotsuga menziesii [mirb.]	Proliferating EM	Extraction buffer	1D-SDS-PAGE	*(in-gel)* LC-MS/MS	4813	SE proteomics	Gautier *et al*., 2018
Pseudotsuga menziesii [mirb.]	Proliferating EM and NEC	Extraction buffer	1D-SDS-PAGE	*(in-gel)* LC-MS/MS	3028 total; 2619 dep	SE proteomics	Gautier *et al*., 2019

2nd STAGE: Development . Zygotic = globular-torpedo transition/ globular to coleoptilar/gymnosperms: after suspensor elongation |Indirect SE = globular-heart-torpedo transition; monocots: late embryo; gymnosperms: after PEM III

Species	Sampled stage	Protein extraction	Separation	MS Identification	Identified elements	Type of analysis	Reference
Cyclamen persicum	Globular (3WAP), torpedo (5WAP) and endosperm	Phenol	2DE	*(in-gel)* LC-MS/MS	62	**ZE proteomics**	Mwangi *et al*., 2013
Cyclamen persicum	Torpedo-staged zygotic and somatic embryos	NA	NA	GC-Ion Trap MS	52 metabolites	**ZE Metabolomics**	Winkelmann *et al*., 2015
Cyclamen persicum	Torpedo zygotic embryo, Globular somatic embryo	Phenol	2DE	*(in-gel)* LC-Q-TOF/MS	23	Comparative	Winkelmann *et al*., 2006
Cyclamen persicum	Torpedo zygotic embryo, globular and torpedo somatic embryos	Phenol	2DE	*(in-gel)* MALDI-TOF/TOF MS	10 of 35 deps	Comparative	Bian *et al*., 2010
Cyclamen persicum	Torpedo-staged zygotic and somatic embryos	Phenol	2DE-DIGE	*(in-gel)* LC- MS/MS	247 proteins out of 300 spots	Comparative	Rode *et al*., 2011
Cyclamen persicum	EC, and globular, torpedo, cotyledonary somatic embryos	Phenol	2DE	*(in-gel)* LC- MS/MS	108 deps (using a proteome reference map)	SE proteomics	Rode *et al*., 2012
Gossypium hirsutum L	Globular and cotyledonary somatic embryos	Phenol	iTRAQ-HPLC	LC-MS/MS	6318	SE proteomics	Ge *et al*., 2015
Theobroma cacao	Torpedo-staged zygotic and somatic embryos	Phenol	2DE	*(in-gel)* LC-MicroTOF-Q MS	32 proteins out of 53 spots	Comparative	Noah *et al*., 2013

3rd STAGE: Maturation. Zygotic: accumulation of storage reserves. SE: cotyledonary stages (establishment of conversion potential)

Species	Sampled stage	Protein extraction	Separation	MS Identification	Identified elements	Type of analysis	Reference
Acer platanoides L	Maturation (14- 22 WAF)	10% (w/v) TCA in acetone containing 20 mM DTT	2DE	*(in-gel)* LC- MS/MS	33 spots; 30 homologued proteins	**ZE proteomics**	Staszak and Pawłowski, 2014
Thlaspi arvense L	Maturation of zyotic embryo (11, 13, 15,17 19 and 21 DAP)	Boiling water	-	GC/MS and LC-MS/MS	112 metabolites	**ZE metabolomics**	Tsoogtbatar *et al*. 2015
Theobroma cacao	zygotic embryos: torpedo (14WAP), early cotyledon (16,18WAP), mature (20WAP). Late-torpedo in SE	Phenol	2DE	*(in-gel)* LC- Q-TOF MS	49 proteins, 72 spots	Comparative	Niemenak *et al*., 2015
Carica papaya	Somatic embryos maturation	Methanol chloroform	-	LC-MS/MS	522	SE proteomics	Almeida *et al*., 2019
Triticum aestivum L. cultivars a) Jinhua 9 and b) Zhongmai 175	Grain maturation (16, 21, 26, 32 and 37 DPA)	TCA-acetone	2DE-DIGE	*(in-gel) MALDI-TOF/TOF MS*	Deps identified: a) 138, b) 127	**ZE proteomics**	Cao *et al*., 2016
Oryza sativa	Late morphogenesis and maturation	Acetone	iTRAQ-SCX	MALDI-TOF/TOF MS	2165 identified proteins, 1744 quantified	**ZE quantitative proteomics**	Zi *et al*., 2013
Phoenix dactylifera L	Mature zygotic (23 WAP) and somatic embryos	TCA-acetone-phenol	2DE	*(in-gel)* MALDI-TOF/TOF MS	23 proteins, 63 spots	Comparative	Sghaier-Hammami *et al*., 2009a
Saccharum spp.	Maturation of EC and NEC	TCA-acetone	RP UPLC	nanoUPLC-ESI-HDMS[E] MS	1267	SE proteomics	Heringer *et al*., 2015
Saccharum spp.	Maturation of somatic embryos	Extraction buffer	Liquid chromatography	LC-MS/MS	2611	SE proteomics	Reis *et al*., 2016

Phoenix dactylifera L	Maturation (on ABA and Sucrose)	TCA–acetone–phenol	2DE	*(in-gel)* MALDI-TOF/TOF MS	28	SE proteomics	Sghaier-Hammami *et al*., 2010
Araucaria angustifolia	Mature zygotic embryo vs torpedo stage	TCA-acetone	2DE	*(in-gel)* MALDI-TOF MS	8 differentially expressed polypeptides	**ZE proteomics**	Silveira *et al*., 2008
Araucaria angustifolia	Mature and germinated zygotic embryo	Acetone-chloroform	1-DE, 2-DE	LC-MS/MS	32 in mature, 24 in germinated	**ZE proteomics**	Balbuena *et al*., 2011
Cunninghamia lanceolata	Cotyledonary zygotic embryos	a) TCA-acetone, b) SDS extraction/acetone precipitation, c) Phenol/methanol-ammonium acetate	2DE	*(in-gel)* LC-MS/MS	a) 40, b) 47, c) 83	**ZE proteomics**	Zhen and Shi, 2011
Pinus pinaster	Cotyledonary-staged somatic and zygotic embryos	Phenol	2DE	*(in-gel)* LC-MS/MS	23 significant proteins overaccumulated in both embryos	Comparative	Morel *et al*., 2014b
Pinus pinaster	Cotyledonary somatic embryos	Phenol	2DE	(in gel) LC-MS/MS	56 proteins, 1428 spots	SE proteomics and ABA determination by	Morel *et al*., 2014a
Picea abies	Mature zygotic embryos	NA	NA	GC-TOF/MS	97 compounds	SE metabolomics	Businge *et al*., 2013
Picea glauca	Maturation of somatic embryos	NA	NA	NMR spectroscopy	35 compounds	SE metabolomics	Dowlatabadi *et al*., 2009
DEVELOPMENTAL SERIES							
Jatropha curcas	Development at 5, 10, 15, 20, 25 and 30 DAF	TCA	2DE	*(in-gel)* MALDI-TOF/TOF MS	104 deps	**ZE proteomics**	Liu *et al*., 2013
Gossypium hirsutum L	Explant, NEC, EC and somatic embryos (globular, torpedo and cotyledonary -staged)	Phenol	2DE	(in gel) MALDI-TOF/TOF MS	155 deps	SE proteomics	Zhou *et al*., 2016
Silybum marianum L.	NEC, PEM, globular or cotyledonary somatic embryos, plantlet germination	NA	NA	TOF MS	20 metabolites	SE metabolomics	Khan *et al*., 2015
Quercus suber L.	Proliferative, cotyledonary and mature somatic embryo	Phenol-ammonium acetate-acetone	2DE-DIGE	*(in-gel)* MALDI-MS/MS	44 proteins,66 spots	SE proteomics	Gomez-Garay *et al.*, 2013
Phoenix dactylifera L	Morphogenesis (12, 14, 17 WAP), maturation (23 WAP) and germination	TCA-acetone-phenol	2DE	*(in-gel)* MALDI-TOF/TOF MS	21 proteins, 194spots	**ZE proteomics**	Sghaier-Hammami *et al*., 2009b
Musa spp	1) IMFB, NEC and EC (2) somatic embryos 0, 30, 45 and 60 days-staged and (3) Gse and non-Gse	Acetone	2DE	*(in-gel)* MALDI TOF-TOF/MS	70 in total; 16 unique for EC, 17 deps	SE proteomics	Marimuthu *et al*., 2019
Elaeis guineensis Jacq.	callus, globular, torpedo and cotyledonar SE and plantlet	Acetone	Phosphoprotein enrichment	LC-MS/MS	460 phosphoproteins	SE phospho-proteomics	Aroonluk *et al*., 2020
Araucaria angustifolia	proembryo, globular, torpedo, early-cotyledonary, late-cotyledonary and mature	TCA-acetone	2DE	LC-MS/MS	96 proteins	**ZE proteomics**	Balbuena *et al*., 2009
Picea abies	PEM proliferation, embryo differentiation (early and late), maturation	NA	NA	GC/TOF MS	52 compounds	SE metabolomics	Businge *et al*., 2012

days after pollination (DAP); differentially accumulated proteins (dap); differentially expressed proteins (dep); germinating somatic embryos (Gse); non-embryogenic calli (NEC); not applicable information (NA); primary embryogenic calli (PEC); synchronous precursor selection (SPS);weeks after flowering (WAF).

†the type of mass spectrometry analysis carried out; we indicate in parenthesis the experiments that based the MS identification in the “in gel” digestion of the spots visualized.

‡ indicates the number of proteins (or metabolites) that had a match in the databases, which is usually different to the total number of proteins quantified or spots visualized.

Supplementary Table 2. Experimental data reported in the papers reviewed about somatic embryogenesis

dicots	monocots	gymnosperms

1st STAGE: Ontogeny . ZE = early stages (before globular). Direct SE= induction; indirect SE = induction and proliferation (monocots: induction to early embryogenesis (embryogenic phase); gymnosperms: EC to PEM)

Species	Sampled stage	Explant, starting material	Media formulation and conditions	Reference
Gossypium hirsutum L	NEC, PEC, globular somatic embryo	Hypocotyls	MSB containing 0.46 µmol/L kinetin and 0.45 µmol/L 2,4-D. NEC were maintained in MSB medium for 6 weeks at 28 ◦C , 16/8 h light/dark photoperiod, were subcultured in fresh MSB medium without hormones. After 3–4 weeks of growth, the somatic-to-embryogenic transition progressed to induction of PEC and globular structures.	Guo *et al*., 2019
Gossypium hirsutum L	NEC, PEC, globular somatic embryo	Hypocotyls	MSB containing 0.46 µmol/L kinetin and 0.45 µmol/L 2,4-D. Calli were subcultured in MSB medium without PGRs	Guo *et al*., 2019b
Medicago truncatula	Induced and proliferating calli	Leaflets	Induction medium:MS salts and vitamins supplemented with 3% (w/v) sucrose, 0.4 µM 2,4-D, 0.9 µM zeatin, 0.2% (w/v) gelrite. Proliferation medium: MS salts and vitamins with 3% (w/v) sucrose and solidified with 0.2% (w/v) gelrite.	Almeida *et al*., 2012
Nothapodytes nimmonianc	NEC and EC	Mature zygotic embryo	Callus subculture: MS medium with 2,4-D + BAP (9.04 + 4.44 µM); then induction of pinkish EC: a) medium devoid of PGRs, or b) medium with BAP + 2,4-D (4.44, 6.66 or 8.88 + 2.26 µM), which resulted in the induction of globular embryos.	Isah and Umar, 2019
Persea americana	NEC and EC	Immature zygotic embryos	Induction: MS medium with 30 g/L sucrose, 4 mg/L thiamine HCl 4, 100 mg/L myo-inositol, 0.41 µM picloram, 3 g/L gellan gum; pH 5.7, in darkness at 25 ºC. Subculturing every two weeks on fresh medium over seven months.	Olivares-García *et al*., 2020
Brachypodium distachyon	EC	Immature zygotic embryos	MS medium, 4 g/L EDTA-Fe, 30 g/L sucrose, 2.5 mg/L 2,4-D, 0.6 mg/L CuSO4, 2 g/L Phytagel, pH 5.8, 25 ºC in the dark	Mamedes-Rodrigues *et al*., 2018
Boesenbergia rotunda	EC	Shoot base meristem	Induction: MS medium with 1 mg/L NAA, 1 mg/L IAA, 30 g/L sucrose and 2 g/L Gelrite. Propagation: MS containing 30 g/L sucrose, 2 g/L Gelrite, and various concentrations of 2,4-D.	Ng *et al*., 2016
Elaeis guineensis Jacq.	1) explant, 2) 14 days of induction, 3) primary callus and 4)pro-embryogenic callus	Zygotic embryos from mature seeds	Induction: MS medium supplemented with salts and vitamins, 20 g/L sucrose, 0.5 g/L glutamine, 2.5 g/L activated charcoal, 450 µM 4-amino-3,5,6-trichloro picolinic acid (Picloram), and solidified with 2.5 g/L Phytagel (Sigma®).	Silva *et al*., 2014
Elaeis oleifera, E. guineensis	Induction stage	Leaves	MS culture solid medium (2.5 g/L Phytagel) supplemented with 30 g/L sucrose, 0.5 g/L of glutamine, 0.5 g/L of casein, 2.5 g/L activated charcoal, and 450 µM of picloram.	Ribeiro *et al*., 2019
Musa spp.	EC and NEC induction	IMFBs	Induction: MA1, medium with 4 mg/L 2, 4-D, 1 mg/L 3-IAA, 1 mg/L NAA and 30 g/L sucrose , pH 5.8, 2 g/L CleriGel. In darkness at 25 °C for 3 months. After 3–6 months of incubation, EC and NEC were observed.	Kumaravel *et al.*, 2017
Zea mays	EC induction	Immature zygotic embryos from inbred lines	Induction: Modified N6 medium, in darkness at 28 °C. Immature embryos induced for 0 d were used as the control.	Ge *et al*., 2017
Zea mays	EC and NEC	Immature zygotic embryos from inbred lines	Induction: N6 medium supplemented with 2 mg/L 2,4-D, 600 mg/L proline, 500 mg/L casein hydrolysate, 200 mg/L L-aspartic acid, 3% sucrose and 5.8g/L agar; pH 5.8. 24 ºC in the dark. Subculture medium: induction medium with 6.5 g/L D-mannitol	Liu *et al*., 2018
Zea mays	EC and NEC	Immature zygotic embryos from inbred lines	Induction: N6 medium supplemented with 2 mg/L 2,4-D, 700 mg/L proline, 450 mg/L casein hydrolysate, 100 mg/L myoinositol, 230 mg/L aspartic acid, 20 g/L mannitol, 3 % sucrose and 7.5 g/L agar; pH 5.8, 26 ºC in the dark. Primary calli sub-culture every 2 weeks into fresh induction medium. After sub-culturing four times, the EC and NEC appeared.	Sun *et al*., 2013
Zea mays	EC and NEC	Immature zygotic embryos from inbred lines	Induction: N6 medium supplemented with N6 salts, 2% sucrose, 25 mmol/L proline, 1 mg/L 2,4-D, 100 mg/L casein hydrolysate, and 1 mg/L N6 vitamins, 3 g/L Gelrite, pH 5.8; addition of 10 mg/L silver nitrate after sterilization. Maintenance of primary callus: N6 medium without silver nitrate. EC maturation: N6 medium with 1 mg/L N6 vitamins, 6% saccharose, 1 mg/L 2,4-D, 1.4 g proline, 100 mg/L casein hydrolysate and 3 mg/L Gelrite; in the dark at 28 °C.	Varhaníková *et al.,* 2014
Araucaria angustifolia	EC	Globular-staged zygotic embryos	Induction (PGR-free): BM medium with 1 g/L glutamine , 1 g/L myo-inositol, 0.5 g/L casein hydrolysate and 30 g/L sucrose, 2 g/L Phytagel. PGR-supplemented induction medium: 4 µM 2,4-D, 2 µM BAP, 2 µM KIN-BM4, 2 g/L Phytagel, pH 5.8 in darkness at 22 ± 2 °C. Subcultures after 30 days of induction, every 21 days in solid BM0 or BM2 culture medium.	Fraga *et al*., 2016
Araucaria angustifolia	EC	Embryogenic suspension cultures	Embryogenic suspension cultures in MSG medium. Subculture: fresh MSG liquid medium with 30 g/L sucrose, 1.4 g/L glutamine and 0.1 g/L myo-inositol (pH 5.7, orbital shaker at 100 rpm in the dark at 25 ± 2 °C). MSG medium was supplemented with 10 uM MpsI inhiibitor SP600125. Control medium was inhibitor-free.	Douétts-Peres *et al*., 2019

Picea balfouriana	EC	Zygotic embryos from mature seeds	Induction: solidified half-strength LM medium with 10 µM 2,4-D and 5 µM 6-BAP, 1% sucrose, 500 mg/L glutamine, 1 g/L casein hydrolysate, and 2% Gelrite at 24 ± 1°C in the dark. Proliferation: induction medium with three different concentrations of 6-BAP (2.5 µM, 3.6 µM, and 5 µM); in darkness at 24 ± 1°C. 2-week intervals subculturing of EC (sample collection every 7 days for metabolite extraction). Early embryo differentiation: half-strength LM without PGRs for 1 week. Promotion of late embryo development and maturation: half- strength LM medium + 61 µM ABA, 0.4% active charcoal, 6% sucrose, 500 mg/L glutamine, 1 g/L casein hydrolysate, and 4% Gelrite, in the dark at 24 ± 1°C. Somatic embryos generated from 100 mg of EC tissue with 3–5 cotyledons germinated were counted. Germination: 1/4 strength LM medium with 0.5% active charcoal, 2% sucrose, 500 mg/L glutamine, 500 mg/L casein hydrolysate,and 3% Gelrite at 24 ± 1°C in the light (30 µEm-2s-1, 16 h photoperiod). Germinated SE with elongated root and hypocotyl were counted.	Li *et al*., 2015
Pseudotsuga menziesii [mirb.]	Proliferating EM	Cotyledonary somatic embryos from 1ry and 2ry embryogenic lines	Repetitive cycles of EMs subculture in Glitz initiation/proliferation medium: Litvay medium with 4.5 µM 2,4-D, 2.2 µM BA, 0.087 M maltose, 4 g/L gellan gum; pH 5.8	Gautier *et al*., 2018
Pseudotsuga menziesii [mirb.]	Proliferating EM and NEC	Cotyledonary somatic embryos from 1ry somatic embryos	Initiation medium from 1ry cotyledonary somatic embryos: Glitz medium supplemented with 4.5 µM 2,4-D, 4.4 µM BA, 0.087 M sucrose, 4 g/L gellan gum (darkness, 23 ºC). The developed EM and NEC from the hypocotyl region were sub-cultured every 2 weeks on Glitz proliferation medium wih 4.5 µM 2,4-D, 2.2 µM BA and 0.087 M maltose solidified with 4 g/L gellan gum (darkness, 23 ºC).	Gautier *et al*., 2019

2nd STAGE: Development . Zygotic = globular-torpedo transition/ globular to coleoptilar/gymnosperms: after suspensor elongation |Indirect SE = globular-heart-torpedo transition; monocots: late embryo; gymnosperms: after PEM III

Species	Sampled stage	Explant, starting material	Media formulation and conditions	Reference
Cyclamen persicum	Torpedo zygotic embryo, Globular somatic embryo	Embryogenic suspension culture initiated from PEDCs	Auxin-free medium: MS medium, Fe EDTA full strength, 2 g/L glucose, 250 mg/L peptone, 0.5 mg/L nicotinic acid, 0.1 mg/L thiamine-HCl, 0.5 mg/L pyridoxine- HCl, 100 mg/L inositol, containing either 30 or 60 g/L sucrose. 24ºC at 100 rpm in darkness	Winkelmann *et al*., 2006
Cyclamen persicum	Torpedo zygotic embryo, Globular and Torpedo somatic embryos	Tubers of seedlings	Induction: half-strength MS medium with 3% sucrose, 2 mg/L 2,4-dichlorophenoxy-acetic acid, 0.2 mg/L benzyladenine, 100 mg/L casein, and inositol. EC establishment on growth regulator-free medium of the same composition.	Bian *et al*., 2010
Cyclamen persicum	Torpedo zygotic and torpedo somatic embryos	Embryogenic suspension culture	Basal medium: half-strength liquid MS medium, full strenght FeEDTA, pH 5.5–5.6, 30 g/L sucrose, 2 g/L glucose, 2.0 mg/L 2,4-D and 0.8 mg/L, 2iP. Induction of SE: growth regulator-free medium of the same basal composition with 4 g/L Gelrite; 24º C in darkness.	Rode *et al*., 2011
Cyclamen persicum	EC, and globular, torpedo, cotyledonary somatic embryos	Embryogenic suspension cultures	Basal medium: half-strength liquid MS medium, full strenght FeEDTA, pH 5.5–5.6, 30 g/L sucrose, 2 g/L glucose, 2.0 mg/L 2,4-D and 0.8 mg/L, 2iP. Induction of SE: basal medium, PGR-free, solidified with 4 g/L Gelrite and sucrose (30 or 60 g/L). Cells and developing somatic embryos were harvested after 0, 1, 3, 7, 21 and 28 days of incubation. 28-day-old embryos developed on medium with 30 g/L sucrose were transferred to basal medium, with or without 10 mg/L ABA; the embryos were incubated for further 28 days at 24°C in the dark.	Rode *et al*., 2012
Gossypium hirsutum L	Globular and cotyledonary somatic embryos	Hypocotyls	Induction: MS medium plus B5 vitamins, supplemented with 0.05 mg/L IAA, 0.05 mg/L kinetin, 0.05 mg/L 2,4-D, 25 g/L glucose, 2 g/L gelrite, pH 5.8. EC emergence: MSB medium supplemented with 25 g/L glucose, 2 g/L gelrite, 0.5 g/L MgCl2, 0.16 mg/L kinetin, 0.08 mg/L IAA, pH 6.5; changed monthly, for 4 months. Somatic embryo induction: MSB supplemented with 25 g/L glucose, 2 g/L gelrite, 0.5 g/L MgCl2, 0.08 g/L kinetin, 0.12 mg/L BA, pH 6.8. Concentration of treatments: ABA (0.01, 0.04, 0.2, 0.4, and 2.0 µM), GA (0.28, 1.4, 2.8, 14, and 28 µM), paclobutrazol (0.01, 0.03, 0.1, and 0.33 µM), and JA (0.1, 0.5, 1, 2, and 10 µM).	Ge *et al*., 2015
Theobroma cacao	Torpedo zygotic and torpedo somatic embryo	Immature floral buds	Petals and staminodes (starting material) of immature floral buds were cultured in PCG (primary callus growth) medium supplemented with 250 mg/L glutamine, 100 mg/L myo-inositol, 1 ml/L DKW vitamin stock (100 g/L myo-inositol, 2 g/L thiamine-HCl, 1 g/L nicotinic acid and 2 g/L glycine), 20 g/L glucose, 18 µM 2,4-D and 45.4 nM thidiazuron). Developed callus were transferred to SCG (secondary callus growth) supplemented with 0.5 ml/L DKW vitamin, 20 g/L glucose, 9 µM 2,4-D and 1.2 µM kinetin medium for 14 days. Then, calli were maintained for 21 days in embryo development medium which is a PGR-free medium supplemented with 1 ml/L DKW vitamins, 30 g/L sucrose and 1 g/L glucose.	Noah *et al*., 2013

3rd STAGE: Maturation. Zygotic: accumulation of storage reserves. SE: cotyledonary stages (establishment of conversion potential)

Species	Sampled stage	Explant, starting material	Media formulation and conditions	Reference
Theobroma cacao	Late-torpedo staged somatic embryo	Secondary somatic embryos from EC	Embryo development medium: DKW salts, vitamins (0.1 g/L myo-inositol, 2 mg/L thiamine–HCl, 1 mg/L nicotinic acid and 2 mg/L glycine), 30 g/L saccharose and 1 g/L glucose. Bioreactor cultures were kept at 28 ± 1 ◦ C in the dark	Niemenak *et al*., 2015
Carica papaya	Somatic embryos maturatic	Mature zygotic embryos	Induction: MS medium with 20 µM 2,4-D, 30 g/L sucrose, 2 g/L Phytagel, pH 5.8, in the dark at 25 ºC. Maturation: MS medium with 0.55 mM myo-inositol, 30 g /L sucrose and 60 g/L PEG at 25 ± 1 °C in the dark for 7 days, under a 16-h photoperiod.	Almeida *et al*., 2019

Phoenix dactylifera L	Mature zygotic (23 WAP) and somatic embryos	Leaves	Induction: MS solid medium, Fe-EDTA, 50 g/L sucrose, 0.1 g/L myo-inositol, 2 mg/L glycine, 0.1 g/L glutamine, KH2PO4, adenine, 0.5 mg/L of 2,4-D, in darkness at 28 ºC. Establishment of embryogenic suspensions: induction medium but liquid with 1 mg/L of 2,4-D, 30 g/L sucrose and activated charcoal, at 120 rpm, 28ºC, 16/8-h (light/dark) period. Somatic embryo maturation: same composition media in agar, without 2,4-D nor activated charcoal, and sucrose at 50 g/L	Sghaier-Hammami *et al*., 2009a
Saccharum spp.	Maturation of EC and NEC	Shoot apical meristem	Induction and proliferation: MS medium supplemented with 20 g/L sucrose, 2 g/L Phytagel, 10 µM 2,4-D, pH 5.8, 25°C in the dark. Maturation: MS medium with 20 g/L sucrose, 2 g/L Phytagel and several concentrations of activated charcoal (0.0, 0.75, 1.5 and 3.0 g/L). After 7 days, photoperiods of 16 h light (60 µmol.m2.s-1) were used for up to 28 days of culture.	Heringer *et al*., 2015
Saccharum spp.	Maturation of somatic embryos	Leaves	Induction: MS medium with 20 g/L sucrose, 2 g/L Phytagel, 10 µM 2,4-D, pH of 5.8; in the dark at 25 °C. After 45 days in culture, induced calli were subcultured every 21 days in the same medium. To test polyamine effects on somatic embryo induction: MS medium supplemented with 30 g/L sucrose and 2 g/L Phytagel, and 0, 10, 100 and 500 µM of putrescine, spermidine, and spermine; kept at 25 °C in the dark for 7 days, then transferred to light for 21 days of culture to generate mature embryos. Regenertion of SE: MS medium with 30 g/L sucrose and 2 g/L Phytagel, in 16 h photoperiod (90 µmol/m2/s) for 30 days.	Reis *et al*., 2016
Phoenix dactylifera L	Maturation (on ABA and Sucrose)	Friable callus	Friable callus were maintained into liquid M3 medium in rotary shaker at 120 rpm, 28 °C under a 16/8-h (light/dark) photoperiod. Proembryonic masses evolved to differentiated SE with weekly subculture in medium M3. At the globular stage (stage 1; 1 month after subculturing) 0.1 g of embryos were transferred to the same culture medium M3 without (control) or with addition of ABA (5, 10, 20 and 40 µM for 4 weeks) or more sucrose (30 and 90 g/L) to the M3 medium (which initially contained of 30 g/L sucrose). Embryos were transferred weekly to fresh medium with or without additives until the structured embryo stage was reached.	Sghaier-Hammami *et al*., 2010
Araucaria angustifolia	Proliferating EC	Immature zygotic embryos of embryogenic cell lines	Induction: BM medium (Gupta and Pullman, 1991) with 5 µM 2,4-D, 2 µM 6-BA, 2 µM kinetin (darkness at 25 ºC). Proliferation: subculture in semi-solid MSG. Maturation medium I: 100 mg (fresh weight) EC on filter papers transferred to basic MSG, solidified with 3 g/L Gelrite®, 1.46 g/L glutamine, 30 g/L sucrose, 3 g/L activated charcoal, 70 g/L maltose, 90 g/L PEG 4000 (in darkness at 25°C). Maturation medium II: maturation medium I with 120 µM ABA.	Jo *et al*., 2014
Pinus pinaster	Cotyledonary somatic and cotyledonary zygotic embryos	Immature zygotic embryos of embryogenic lines	Proliferation medium: mLV supplemented with 3 g/L gellan gum, 0.087 M sucrose, 2 µM 2,4- D, 1 µM benzyladenine (BA). Maturation: mLV without PGR, supplemented with 0.2 M sucrose, 80 µM ABA and 9 g/L gellan gum.	Morel *et al*., 2014b
Pinus pinaster	Cotyledonary somatic embryos	Immature zygotic embryos of embryogenic lines	Proliferation medium: mLV supplemented with 3 g/L gellan gum, 0.087 M sucrose, 2 µM 2,4- D, 1 µM benzyladenine (BA). Maturation: mLV without PGR, supplemented with 0.2 M sucrose, 80 µM ABA and either 4 or 9 g/L gellan gum.	Morel *et al*., 2014a
Picea abies	Mature zygotic embryos	EC from embryogenic cell lines	EC subculture (2-week interval): half-strength LP solid medium with 9.0 µM 2,4-D and 4.4 µM N6-benzaldenine; proliferation of embryogenic structures after 5-8 weeks. Early embryo differentiation from PEMs: half strength LP-medium lacking PGRs. Promotion of late embryo development and maturation: DKM medium with 29 µM ABA, with either 3% (w/v) sucrose or 3% (w/v) maltose and 7.5% (w/v) PEG.	Businge *et al*., 2013
Picea glauca	Maturation of somatic embryos	Mature zygotic embryos	Induction: AE medium with 10 mM 2,4-D, 5 mM BA, 5% (w/v) sucrose, and 0.4% Difco Bacto-agar, pH 5.8. Embryogenic tissues were transferred on maintenance medium: AE medium containing 10 mM 2,4-D, 2 mM BA, and 3% sucrose, pH 5.8. Promotion of embryo development: 1 week in AE medium without PGRs, then, 50 mg of tissue plated on maturation medium: AE medium containing 50 mM ABA and 5% sucrose, 0.8% agar	Dowlatabadi *et al*., 2009
DEVELOPMENTAL SERIES				
Gossypium hirsutum L	Explant, NEC, ECs and somatic embryos (globular, torpedo and cotyledon)	Hypocotyls	Induction: MSB medium with 1.0 mg/L IBA and 0.1 mg/l kinetin. After 40 days, induction of EC: MSB medium containing twice the concentration of KNO3, NH4NO3 free, supplemented with 3% (w/v) glucose, 0.25% (w/v) Phytagel, 0.5 mg/L IBA, 0.15 mg/L kinetin, 1 g/L glutamine, and 0.5 g/L asparagine. Cultures maintained at 28 °C, 14-h photoperiod (irradiance of 135 µmol m-2 s-1).	Zhou *et al*., 2016
Silybum marianum L.	NEC, PEM, globular or cotyledonary somatic embryos, plantlet germination	Petiole explant from in-vitro germinated seedlings	Induction: SH medium with 3 % sucrose (w/v) and 0.8 % (w/v) agar in 150 ml conical flask supplemented with (0.5, 1.5, 2.5, 5.0 or 8.0 mg/L) of 2,4-D or BA alone or 1.5 mg/L BA with 2,4-D (0.5, 1.5, 2.5, 5.0 or 8.0 mg/L), pH 5.8, 16 h photoperiod (40 µmol m-2 s-1) at 25 °C. Control medium was PGR free. After 4 weeks induction, callus producing PEMs were transferred into SH medium containing (0.5, 1.5, 2.5, 5.0 or 8.0mg/L) of 2,4-D or BA alone, or1.5mg/L BA in combination with 2,4-D (0.5, 1.5, 2.5, 5.0 or 8.0 mg/L). Germination medium: half strength MS medium with (0.0, 0.5, 1.5 or 2.0 mg/L) of GA.	Khan *et al*., 2015
Quercus suber L.	Proliferative, cotyledonary and mature somatic embryos	Immature zygotic embryos	Basal culture medium: MS with ascorbic acid, nicotinic acid, glutamine, calcium pantothenate, pyridoxine-HCl and thiamine-HCl, sucrose and 2,4-D, pH 5.7. Induction medium: basal medium, 8 g/L agar and 22.6 mM 2,4-D. Proliferation stage in PGR-free medium. Maturation of cotyledonar embryos: basal culture medium plus 1% activated charcoal.	Gomez-Garay *et al.*, 2013

Musa spp	1) IMFB, NEC and EC (2) somatic embryos 0, 30, 45 and 60 days-staged and (3) Gse and non-Gse	IMFBs	Callus induction medium supplemented with 1 mg/L IAA, 1 mg/L NAA and 4 mg/L 2,4-D, and different concentrations of calcium chloride (10, 15 and 20 mM) for calcium-relatd proteins induction. Incubation in darkess at 25 ± 2 ◦C up to 8 months without subculture. EC and NEC samples were collected in triplicates. Friable PEM of EC were transferred to liquid suspension medium supplemented with 1.1 mg/L 2,4-D and 250 µg/L zeatin. One milliliter of embryonic cell suspension was plated on somatic embryo regeneration medium supplemented with 200 µg/L NAA, 80 µg/L kinetin, and 40 µg/L zeatin; in darkness at 25 ± 2 ◦C. Samples of somatic embryos (at 0, 30, 45 and 60 days after initiation) were collected in triplicates. Well-developed somatic embryos were transferred to germination medium supplemented with 0.5 mg/L BAP and 2 mg/L IAA; fluorescent light of 40.54 µmol m−2 s−1 with a photoperiod of 16/8 (light/dark) at 25 ± 2 ◦C. After 90 days, the Gse (with shoots and with both shoots and roots) and NGses (without shoot and roots) were collected in triplicates. To stimulate endogenous auxin, different concentrations of Trp were added (122.41, 244.82 and 489.64 µM) and IAA concentration was increased from 5.7 to 11.41 µM, 17.12 and 22.8 µM. To induce endogenous cytokinins, adenylate types of cytokinins like BAP (2.21 and 4.43µM) and kinetin (2.32 and 4.64µM) were added separately and in combination (2.21 µM BAP + 2.32 µM kinetin and 4.43 µM BAP + 4.64 µM kinetin).	Marimuthu *et al*., 2019
Elaeis guineensis Jacq.	callus, globular, torpedo and cotyledonar somatic embryos and plantlet	Immature zygotic embryos	Callus induction under dark conditions on N6 medium pH 5.75 containing 2 mg/L of 2,4-D, 30 g/L of sucrose, and 2 g/L of phytagel; subculture every month for 3–5 months until differentiation of globular structures. Establishment: N6 medium with 0.1 mg/L of 2,4-D, 0.16 g/L of putrescine, 0.5 g/L of casein amino acids, 30 g/L sucrose, 2 g/L phytagel, and 2 g/L activated charcoal under cool-white fluorescent lamp (50–60 µmol m−2 s−1) for 16 h photoperiod. Regeneration: small shoot transferred to a modified N6 medium containing 0.5 g/L activated charcoal and 30 g/L sucrose under same photoperiod light condition.	Aroonluk *et al*., 2020
Picea abies	PEM proliferation, embryo differentiation (early and late), maturation	EC from embryogenic cell lines	EC subculture (2-week interval): half-strength LP solid medium with 9.0 µM 2,4-D and 4.4 µM N6-benzaldenine. After 5-8 weeks embryogenic structures started to proliferate. Early embryo differentiation from PEMs: half strength LP-medium lacking PGRs. Promotion of late embryo development and maturation: DKM medium with 29 µM ABA.	Businge *et al*., 2012

1-Naphthaleneacetic acid (NAA); 2,4-dichlorophenoxyacetic acid (2,4-D); 6-benzylaminopurine (BA); abscisic acid (ABA); embryogenic calli (EC); germinating somatic embryos (Gse); gibberellic acid (GA); immature male flower buds (IMFB); Indole 3-acetic acid (3-IAA); jasmonic acid (JA); MS medium plus B5 vitamin (MSB); non-embryogenic calli (NEC); polyethylene glycol (PEG); pre-embryoid masses (PEMs); primary embryogenic calli (PEC); Schenk and Hildebrandt (SH); tryptophan (Trp)
